# Identification of protein biomarkers to differentiate between gram-negative and gram-positive infections in adults suspected of sepsis

**DOI:** 10.1186/s12879-025-11973-5

**Published:** 2025-11-14

**Authors:** Mahnaz Irani Shemirani, Anna-Karin Pernestig, Jens Björkman, Diana Tilevik, Astrid von Mentzer, Mikael Ejdebäck, Anders Ståhlberg

**Affiliations:** 1 Department of Diagnostics and Interventions, Umeå University, Umeå, Sweden; 2 Sahlgrenska Center for Cancer Research, Department of Laboratory Medicine, Institute of Biomedicine, Sahlgrenska Academy, University of Gothenburg, Gothenburg, Sweden; 3 Systems Biology Research Centre, School of Bioscience, University of Skövde, Skövde, Sweden; 4 Formerly: TATAA Biocenter AB, Presently: Independent Scholar , Gothenburg, Sweden; 5 Department of Microbiology and Immunology, Institute of Biomedicine, Sahlgrenska Academy, University of Gothenburg, Gothenburg, Sweden; 6 Wallenberg Centre for Molecular and Translational Medicine, University of Gothenburg, Gothenburg, Sweden; 7 Science for LifeLaboratory, Institute of Biomedicine, University of Gothenburg, Gothenburg, Sweden; 8 Department of Clinical Genetics and Genomics, Sahlgrenska University Hospital, Gothenburg, Region Västra Götaland, Sweden

**Keywords:** Sepsis, Diagnostics, Gram-negative bacteria, Gram-positive bacteria, Machine learning, Proteomics, Biomarkers

## Abstract

**Background:**

Sepsis is mostly caused by bacterial infections and requires a prompt diagnosis. There is a need for improved diagnostics by differentiating between gram-negative and gram-positive bacterial infections.

**Methods:**

The plasma levels of 285 unique proteins in patients with gram-negative infection (*n* = 154), gram-positive infection (*n* = 92), and in healthy controls (*n* = 35) were quantified using proximity extension assay. Three machine learning algorithms; random forest, recursive feature elimination, and adaptive least absolute shrinkage and selection operator (Lasso) were employed to identify discriminative proteins, with their effectiveness assessed using accuracy metrics. The selected proteins were further evaluated for their ability to differentiate between gram-negative and gram-positive infections through logistic regression and area under the receiver operating characteristic curve.

**Results:**

We identified 55 discriminative proteins differentiating between gram-negative and gram-positive infections using the Lasso, the best performing algorithm. The discriminative proteins achieved AUROC values of 0.69 for gram-negative infections and 0.66 for gram-positive infections, both compared to the remaining groups, and 0.58 for differentiating between the two infection groups. Comparative statistical analysis revealed no significant differences in protein expression between gram-negative and gram-positive patients.

**Conclusions:**

We identified 55 proteins with some discriminative potential between gram-negative and gram-positive infections. However, the overall predictive performance was low and did not exceed that of established single biomarkers. These findings highlight the challenges of applying a multimarker approach in infection classification and emphasize the need for further studies using larger and more diverse cohorts, as well as broader analytical methods, to explore their potential clinical utility.

**Clinical trial:**

Not applicable.

**Supplementary Information:**

The online version contains supplementary material available at 10.1186/s12879-025-11973-5.

## Background

Sepsis is a life-threatening condition resulting from a dysregulated response to infection, with the risk of rapid progression to septic shock and potentially fatal outcomes without timely recognition and treatment [1]. Bacterial infection is the major cause of sepsis [1], with both gram-negative and gram-positive bacteria contributing to its development and requiring different empirical treatment approaches [2–4]. Current laboratory techniques for bacterial identification largely depend on blood culture, which is time-consuming and often incompatible with the urgent need for rapid life-saving interventions [5]. Consequently, standard protocols recommend the immediate administration of broad-spectrum antibiotics, ideally within one hour for adults at high risk of septic shock or with a strong suspicion of sepsis [6]. While prompt antibiotic administration is crucial for survival, it can lead to antibiotic misuse or overuse due to the lack of information on the specific bacterial type. Although species-level identification is ideal for guiding therapy, even the early classification of infection as gram-negative or gram-positive can inform initial antibiotic decisions. For instance, clinicians may choose different classes of empiric antibiotics depending on whether the infection is suspected to be caused by gram-negative bacteria—such as *Escherichia coli*, *Klebsiella pneumoniae*, or *Pseudomonas aeruginosa*—or gram-positive organisms, like *Staphylococcus*, *Streptococcus*, or *Enterococcus* given their differing resistance profiles and treatment strategies [7].

This underscores the pressing need for rapid diagnostic tests that can quickly differentiate between gram-negative and gram-positive bacterial infections [8]. Such advancements would enable clinicians to tailor antibiotic therapy more accurately, thus minimizing the reliance on broad-spectrum antibiotics [9], mitigating the risks of resistance and improving patient outcomes [10]. Research has therefore focused on developing culture-independent diagnostic methods, including those utilizing blood-based markers, to address this challenge [11–14].

To date, more than 250 biomarkers have been proposed for diagnosis of sepsis [15]. Most of them can be used in risk stratification and to assess treatment progress and prognosis of sepsis [15]. Only a limited number of biomarkers are currently used individually for diagnosing sepsis in clinics, such as C-reactive protein (CRP) and procalcitonin. These biomarkers have limitations in sensitivity and specificity, with CRP exhibiting a sensitivity range of 68–92% and a specificity of 40–76%, and procalcitonin showing a sensitivity of 0.75% and specificity of 0.79%. [12, 16, 17]. Several studies have suggested different patterns of blood markers in patients with gram-negative and gram-positive bacterial sepsis [18–22], such as interleukin-10 (IL-10) and interleukin-8 (IL-8) being reported as elevated in gram-negative bacterial compared to gram-positive sepsis [19, 20]. While these findings represent promising advances, depending on a single biomarker may lack the specificity or sensitivity required to assess sepsis adequately, given its complexity in involving multiple body processes. In this context, analyzing serological alterations in a set of blood markers may offer a more accurate classification of the type of bacteria causing the disease.

The plasma levels of proteins are often determined using mass spectrometry (MS) [23–27]. However, MS has limitations in quantifying a broad range of concentrations for various plasma proteins [28]. Targeted approaches based on antigen detection, such as the proximity extension assay (PEA), are gaining popularity in plasma proteomics [29, 30] as they have a broader detection range and thus can detect more proteins compared to MS [28].

In this study, we conducted protein profiling using the PEA to target proteins from four Olink panels (Olink Bioscience, Uppsala, Sweden) [31]. Our aim was to assess the capability of proteins included in these panels as differentiating markers in classifying gram-negative and gram-positive infections in patients suspected of having sepsis. We employed three supervised machine learning methods and selected discriminative proteins based on the algorithm that demonstrated the highest accuracy. Since the effectiveness of the selected proteins as differentiating markers needed to be confirmed, we predicted the classes and evaluated their predictive performance. The reliability of these discriminative proteins was further assessed through internal validation.

## Methods

### Study design

Data and samples originated from a prospective observational study of community-onset severe sepsis and septic shock in adults conducted at Skaraborg Hospital, Sweden from September 2011 to June 2012 [32]. The study was approved by the Regional Ethical Review Board of Gothenburg (376–11) and only included participants who gave their written informed consent.

The sampling for laboratory data was performed at the time of admission to the emergency department according to routine hospital procedures and prior to the administration of antibiotic therapy. To identify bacterial species, microbiological culturing followed by MALDI TOF MS was performed as previously described [33]. All medical records were retrospectively reviewed by two senior specialists in infectious diseases to determine whether the patients fulfilled Sepsis-3 criteria. Following the review, it was found that not all patients met the criteria for sepsis. According to Sepsis-3 criteria, bacterial sepsis was defined as bacterial infection-induced organ dysfunction characterized by a rise in total SOFA ≥2 [34, 35]. Verified bacterial infection was defined as a clinical infection with identification of relevant bacteria by culture or typical clinical symptoms, such as erysipelas. The patients included in the current study were those with a verified bacterial infection. Healthy control samples in the current study were obtained from blood donors who provided consent for their samples to be used in research. At the time of sampling (2014), ethics approval was not required for consented individuals. For protein quantification, blood samples were collected using sodium citrate tubes, and plasma was stored at − 80 °C until subsequent analysis.

### Protein quantification

Protein quantification was conducted using PEA at TATAA Biocenter, Gothenburg, Sweden following the manufacturer’s instructions. Four Olink biomarker panels (Olink Bioscience, Uppsala, Sweden) [31]—Cardiometabolic (CM) (v.3603), Cardiovascular II (CVD II) (v.5004), Immune Response (IR) (v.3202), and Inflammation (Inf) (v.3021)—were employed to analyze proteins in the plasma samples collected from 291 patients with bacterial infection, including 184 with gram-negative infection and 107 with gram-positive infection, along with samples from 40 healthy control individuals.

Sample readouts failing the internal quality assessment based on the manufacturer’s instruction were excluded from further analysis. In total, 50 samples were excluded: 11 failed quality control in two panels, and 39 in one panel. This resulted in 281 valid samples, comprising 154 from patients with gram-negative infection, 92 from patients with gram-positive infection, and 35 samples from healthy control individuals. Among the gram-negative samples, 78 were identified as *Escherichia coli*, 31 as *Haemophilus influenzae*, 20 as *Klebsiella pneumoniae*, 12 as *Pseudomonas aeruginosa*, 10 as *Proteus* spp., and 3 as *Mycoplasma pneumoniae*. Among the gram-positive samples, 30 were *Streptococcus pneumoniae*, 27 *Staphylococcus aureus*, 23 *Clostridioides difficile*, 8 Group A streptococci (*Streptococcus pyogenes*), and 4 Group B streptococci (*Streptococcus agalactiae*). Patient characteristics stratified by gram-positive and gram-negative infections are presented in Table 1.

**Table 1:** Patient characteristics

Characteristic	All patients (n = 246)	Result gram-staining		*p*** value**
		Gram-positive (n = 92)	Gram-negative (n = 154)	
Age (years)	76 (65–85)	70 (58–84)	78 (69–86)	0.004
Sex (female)	116 (47.2)	34 (37.0)	82 (53.2)	0.01
***Focus of infection***^a^				
Respiratory	73 (29.7)	36 (39.1)	37 (24.0)	< 0.001
Abdominal	15 (6.1)	7 (7.6)	8 (5.2)	< 0.001
Urogenital	103 (41.9)	13 (14.1)	90 (58.4)	< 0.001
Bones/soft tissue	10 (4.1)	8 (8.7)	2 (1.3)	< 0.001
Other	43 (17.5)	27 (29.3)	16 (10.4)	< 0.001
***Clinical data***				
CRP (mg/L)	122 (57–216)	121 (55–222) [*n* = 90]	125 (58–209) [*n* = 153]	0.62
Temperature (°C)	38.0 (37.0–38.7)	38.2 (37.3–38.8) [*n* = 82]	37.9 (37.0–38.7) [*n* = 138]	0.14
P-lactate (mmol/L)	1.9 (1.4–2.9)	2.1 (1.4–3.3) [*n* = 88]	1.8 (1.4–2.7) [*n* = 149]	0.17
LPK (x10^9^ cells/L)	12.8 (9.1–16.4)	13.8 (10.1–17.2) [*n* = 91]	12.6 (8.4–16.1) [*n* = 153]	0.07
ICU (yes)	19 (7.7)	5 (5.4)	14 (9.1)	0.30

Data presented as median (Q1–Q3) or number (percentage). Complete clinical data were not available for all patients, in such cases, the exact number is given in square brackets. *p* values refer to comparisons between patient groups with gram-positive and gram-negative bacterial isolates. Differences between groups were assessed by Chi-square test, Fisher´s exact test, or Mann Whitney U test, as appropriate. CRP; C-reactive protein; ICU, intensive care unit; LPK, leukocyte particle concentration

^a^Data on focus of infection were inferred from ICD-10 diagnosis codes, which were available for 244 patients

Among the 368 analyzed proteins, nine proteins were quantified in duplicate, and one protein (interleukin-6, IL6) was quantified in triplicate due to assay overlap between panels (Additional file 1) which were averaged. The PEA readout was converted to a normalized protein expression (NPX) value on the log2 scale, where a high NPX value corresponds to high protein abundance. Data for the different panels were combined according to the manufacturer’s instructions.

### Missing not at random handling

Utilizing the PEA, each assessed protein is associated with a specific limit of detection (LOD), defined as three standard deviations over the background signal. We treated protein values below the LOD as missing not at random (MNAR) [36]. The distribution of proteins, concerning the amount of missing data, is illustrated in Additional file 2.

To determine the impact of proteins with varying degrees of MNAR, we generated ten datasets with missing data cutoffs of less than 5, 10, 20, 25, 30, 40, 50, 60, 70, and 80% per sample group (Additional file 3). Subsequently, the missing data were imputed within groups using GSimp (https://github.com/WandeRum/GSimp) [37]. The imputation was executed in R v.4.1.1 [38] with default parameters. Notably, for the proteins assessed multiple times, imputation was carried out prior to averaging. The optimal missing data threshold was then determined based on the accuracy metric of three feature selection algorithms, contributing to the precision of our imputation approach and subsequent analyses.

### Overview of the feature selection model

To identify the proteins that optimally separate gram-negative and gram-positive infection using healthy controls as a reference, we tested three supervised algorithms commonly used for feature selection; random forest (RF) [39], least absolute shrinkage and selection operator (Lasso) [40], and recursive feature elimination (RFE) [41]. Each feature selection method was applied to the ten datasets with different degrees of missing data and the prediction accuracy of the classifiers was assessed. To evaluate the predictive performance of the algorithms, each dataset was randomly divided into a training and a test dataset with 80% and 20% of the samples in each group respectively, ensuring reproducibility in the split. A brief description of each algorithm is provided below.

#### Random forest

Random forest generates classification trees based on samples and outputs the most selected group by the trees in a classification question [42]. We generated RF models using 100 decision trees where each tree was built on the bootstrap samples from the training set. Gini impurity was selected as a quality measure of a decision. The number of samples in each sample group was unbalanced, hence we specified cost-sensitive learning to adjust for this imbalance. The number of features randomly selected at each decision point of the training set was explored separately in each dataset, ranging from 1 to 20. To evaluate the optimal number of features we performed ten-fold cross-validation of three repeats on whole data and determined the accuracy scores and standard deviations (std). The number of features with the highest accuracy and lowest std was deemed the most preferred in the decision point of the training set. The RF model prediction was made on the test set and the model classification performance was evaluated using a classification report of precision, recall (or sensitivity), f1-score, and accuracy.

#### Least absolute shrinkage and selection operator

Lasso is a regularization technique that shrinks the magnitude of the redundant or irrelevant predictor’s coefficient to zero [43]. We generated the Lasso model on training sets with a tuned lambda of 0.02, repeating the process 1000 times. The value of the lambda constant was automatically determined by generating the LassoCV model on each dataset and tuning the lambda hyperparameter within the range of 0 to 1 with a step size of 0.01. We utilized ten-fold cross-validation on whole data to discover the optimal lambda parameter and repeated this process three times. Lasso model prediction was made on the test set and the predictive power of the model was evaluated using mean square error (MSE).

#### Recursive feature elimination

Recursive feature elimination is a wrapper algorithm that uses different machine learning algorithms at the core [41]. This quality enables us to explore different algorithms which are aligned with the structure of the data. First, five algorithms including logistic regression (LR), perceptron, gradient boosting, decision tree, and support vector machine were explored for the base core in the RFE algorithm. The accuracy and std of each core were determined after ten-fold cross-validation of the whole data which was repeated three times. Thereafter, an RFE cross-validate model with default parameters was generated on the training set of each dataset using ‘Logistic regression’ (RFE-LR) which provided the highest accuracy among core algorithms. RFE-LR model prediction was made on the test set and the predictive performance of the model was evaluated using the classification report of precision, recall, f1-score, and accuracy.

### Statistical analysis

A comparative analysis was conducted using a student t-test to statistically compare samples across groups. *P*-values were adjusted for false discovery rate using the Benjamini-Hochberg method with a threshold of *p_adj* < 0.05. For unsupervised clustering, we applied principal component analysis (PCA), and *t*-distributed stochastic neighbor embedding (tSNE). Following the selection of predictive proteins, LR and “area under receiver characteristic operator” (AUROC) [44] were employed for the assessment of the predictive performance in classifying the three groups of participants. All statistical tests were performed in Jupyter Notebook v.6.0.3 [45] using the Scikit-Learn library [46] and default settings.

## Results

### Optimizing protein biomarker selection

**Fig. 1:**
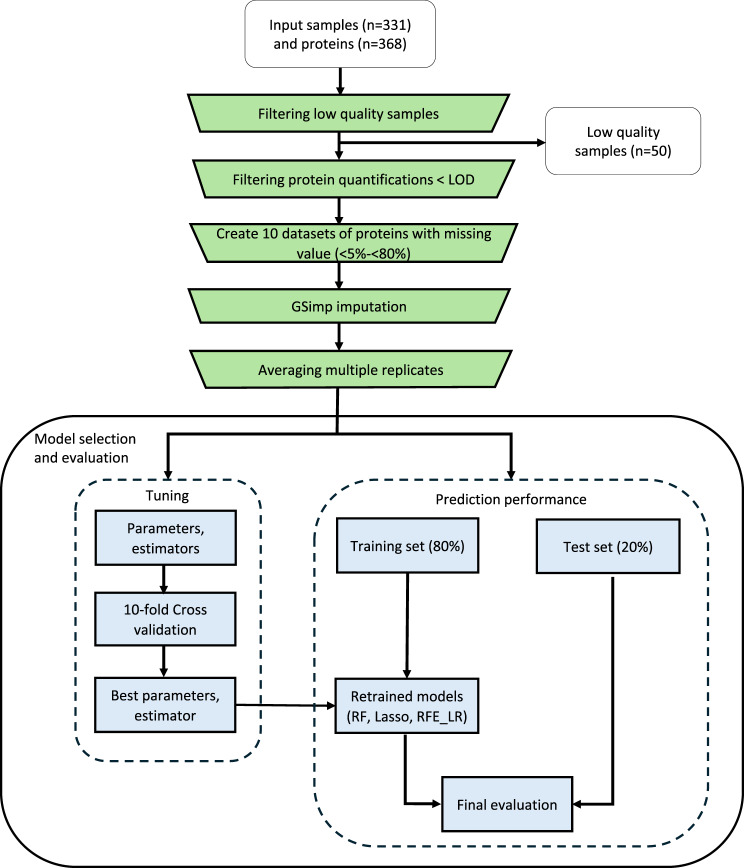
Workflow of processing data. Preprocessing (green boxes), selection, and evaluation of model algorithms (blue boxes). RF, random forest; Lasso, least absolute shrinkage and selection operator; RFE-LR, recursive feature elimination-logistic regression

To identify protein biomarkers distinguishing between gram-negative and gram-positive infections we analyzed 368 proteins by proximity extension assay (PEA) in plasma samples from patients with verified bacterial infection and from healthy controls. The data was processed as illustrated in Fig. 1 through quality control, missing data handling, imputation, model selection, and evaluation.

**Fig. 2:**
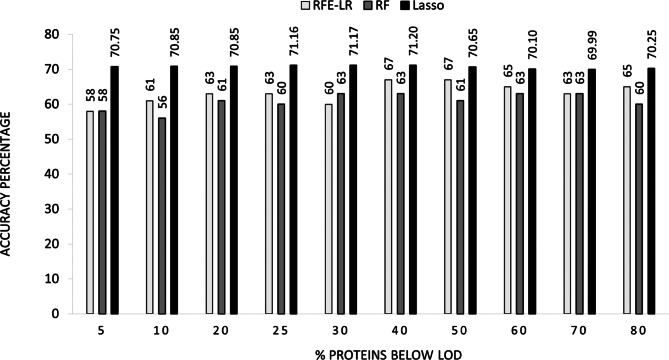
Evaluation of model performance. Accuracies of the three models were determined using a hold-out test on datasets with missing data values below LOD ranging from < 5% to < 80%. RFE-LR (white bar), RF (light grey bar) and Lasso (dark grey bar)

Among all proteins, 253 displayed less than 5% missing data, while 319 had less than 80% missing data in either sample group (Additional file 3). The assessment of incorporating proteins with more than 5% missing data, utilizing datasets with missing cutoffs ranging from 5% to 80% and three different feature selection models, revealed that RF, Lasso, and RFE-LR offered the best accuracies of 63.0%, 71.2%, and 67.0%, respectively, when considering proteins with less than 40% missing data (Fig. 2). This corresponded to 285 relevant unique proteins for downstream analysis (Additional file 3). Precision, recall, and f1-score comparisons between the two models, RF and RFE-LR, further confirmed optimal predictive performance when utilizing proteins with less than 40% missing data. Detailed results of the comprehensive comparative analysis of the methods are presented in Additional file 3.

In an overall comparison of the methods across 10 datasets to identify the best-performing algorithm, Lasso achieved an average classification accuracy of 70.7%, outperforming the machine learning models RF and RFE-LR, which achieved average accuracies of 60.8% and 63.2%, respectively.

### Protein characteristics and sample distribution pattern

To compare protein levels across the three groups (patients with gram-negative infection vs gram-positive infection vs healthy controls), we conducted student t-test with Benjamini-Hochberg adjustment of 285 proteins. This analysis indicated no significant differences in the expression of individual proteins between patients with gram-negative and gram-positive infections, with CCL28 showing the smallest adjusted *p*-value of 0.29. Comparison of samples from patients with gram-negative or gram-positive infections to healthy controls revealed significant differences in protein expressions, serving as a control to demonstrate expected variations between two fundamentally distinct conditions. Specifically, 284 out of 285 proteins for gram-negative infections and 214 out of 285 for gram-positive infections displayed statistically significant differences (Additional file 4).

**Fig. 3:**
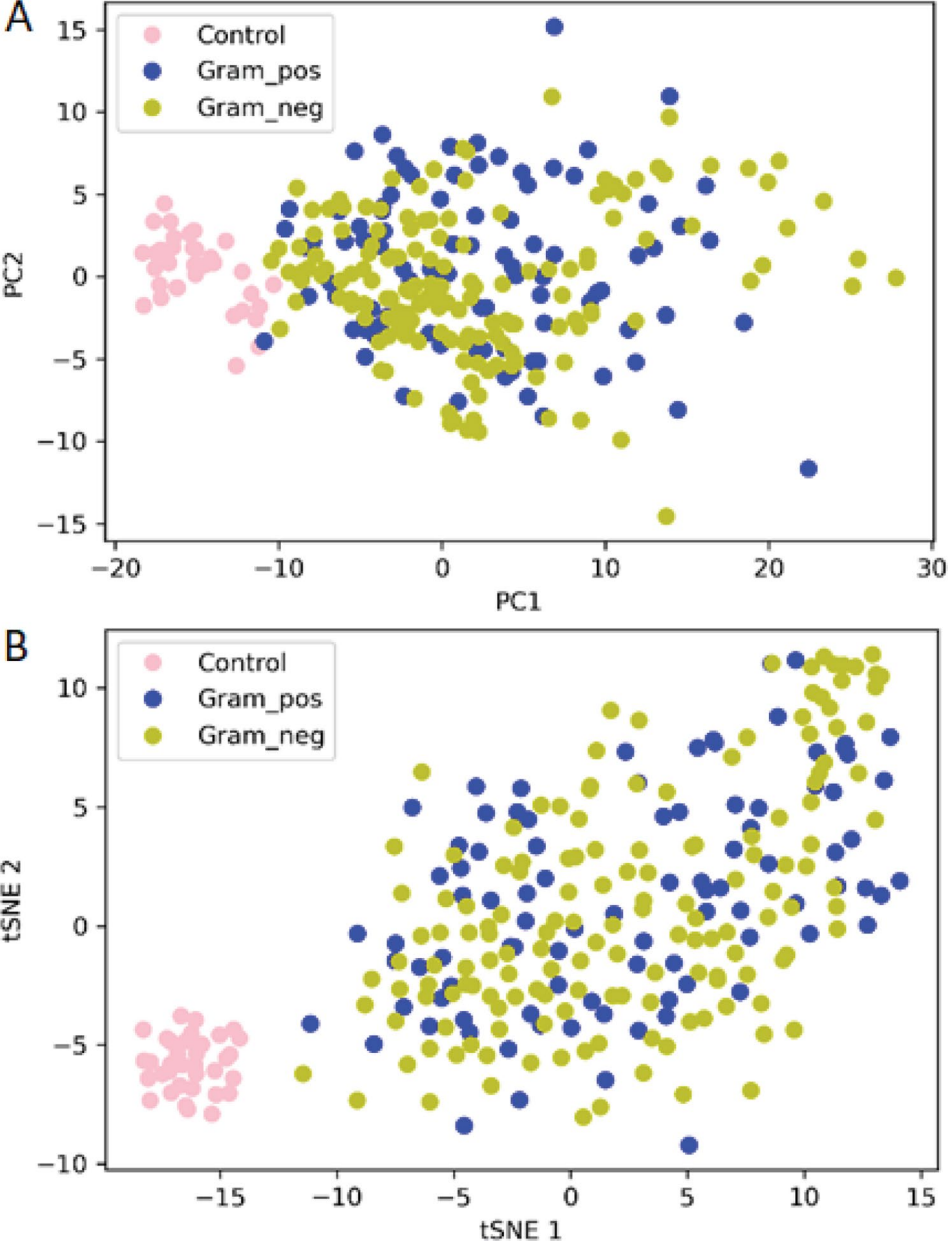
Distribution of samples from patients using 285 proteins. **A**) Principal component analysis (PCA) plot **B**) *t*-distributed stochastic neighbor embedding (tSNE) plot. Yellow dots: samples from patients with gram-negative infection, blue dots: samples from patients with gram-positive infection, pink dots: samples from healthy control individuals

To visualize the distribution of samples in a multidimensional space, we employed unsupervised learning of PCA and tSNE (Fig. 3). Both methods revealed a distinct separation of samples from patients with gram-negative and gram-positive infection from those of healthy control individuals, with a noticeable overlap between the two groups of patients, suggesting similarities in the molecular profiles of these two infection types.

### Discriminative proteins for bacterial typing

To identify the most discriminative protein biomarkers from the pool of 285, we employed Lasso regression to construct a model predicting the distinct groups: patients with gram-negative or gram-positive infection, along with healthy control individuals (refer to Materials and Methods). The Lasso regression model identified 55 proteins with non-zero coefficients, comprising 33 proteins with positive and 22 with negative coefficients (Additional file 5). Notably, the ‘Inf’ and ‘CVD II’ panels exhibited the highest protein inclusion rates, with 24 and 19 proteins, respectively. Additionally, the ‘CM’ panel featured ten proteins, while the ‘IR’ panel included seven.

**Fig. 4:**
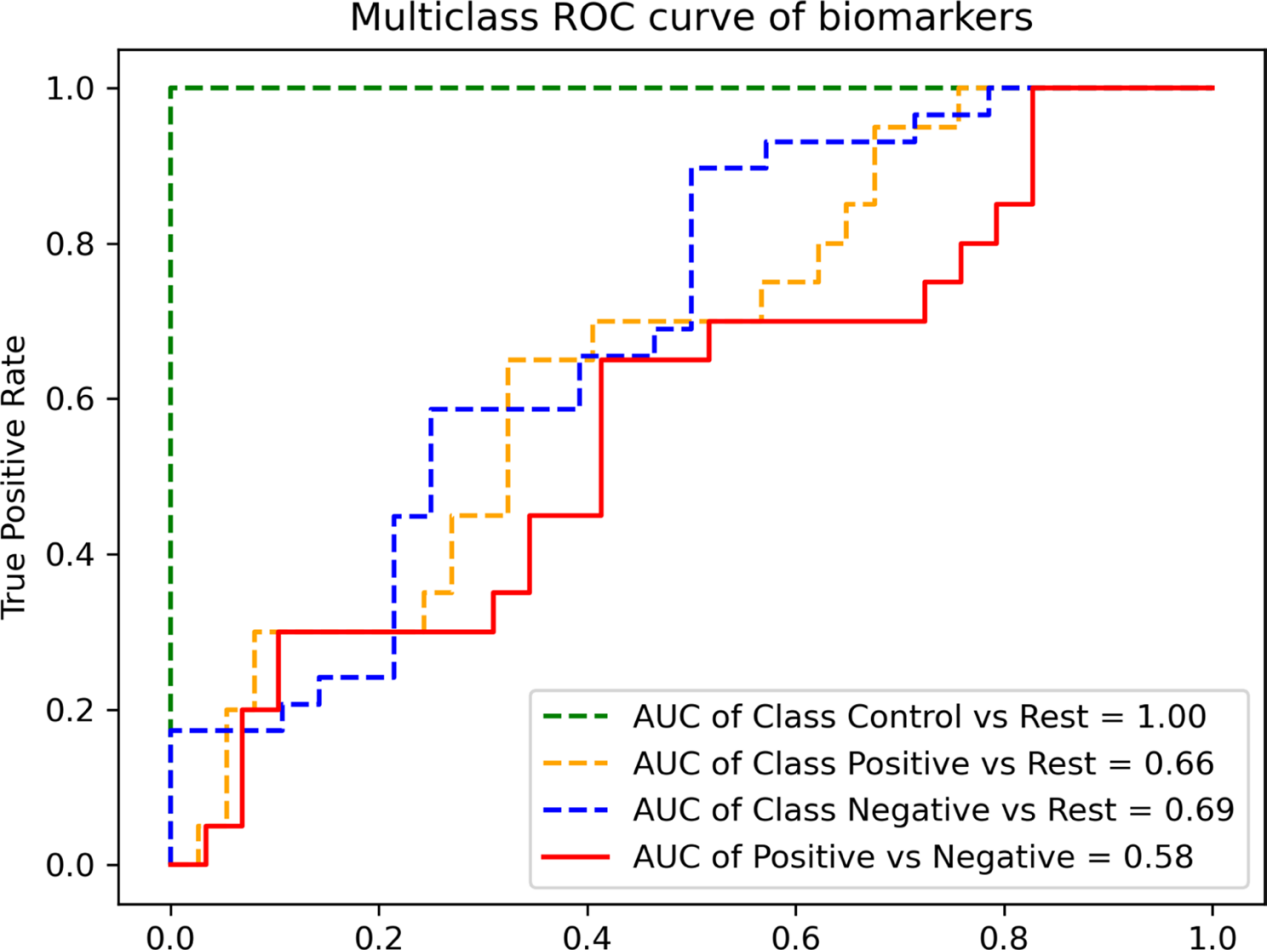
The predictive power of the 55 discriminative proteins in classifying the groups of participants. Blue dashed line: patients with gram-negative infection, yellow dashed line: patients with gram-positive infection, green dashed line: healthy control individuals, red line: direct comparison between patients with gram-positive and gram-negative infections, excluding healthy controls

To assess the performance of the discriminative proteins, an LR model was trained using the 55 selected proteins, and the AUROC metric was employed (Fig. 4). The 55-protein model demonstrated high performance with an area under the curve (AUC) of 1.0 (95% CI 1, 1) for distinguishing samples from both gram-negative and gram-positive infections from those of healthy controls. Specifically, the model exhibited an AUC of 0.69 (95% CI 0.586, 0.794) for discriminating patients with gram-negative infection from the rest, and for gram-positive infection data, it achieved an AUC of 0.66 (95% CI 0.549, 0.771) in distinguishing the group from the rest.

The model performance for discriminating between the two groups of gram-negative and gram-positive infections yielded an AUC of 0.58 (95% CI 0.505, 0.655), indicating no clear distinction between the two infection types (Fig. 4).

**Fig. 5:**
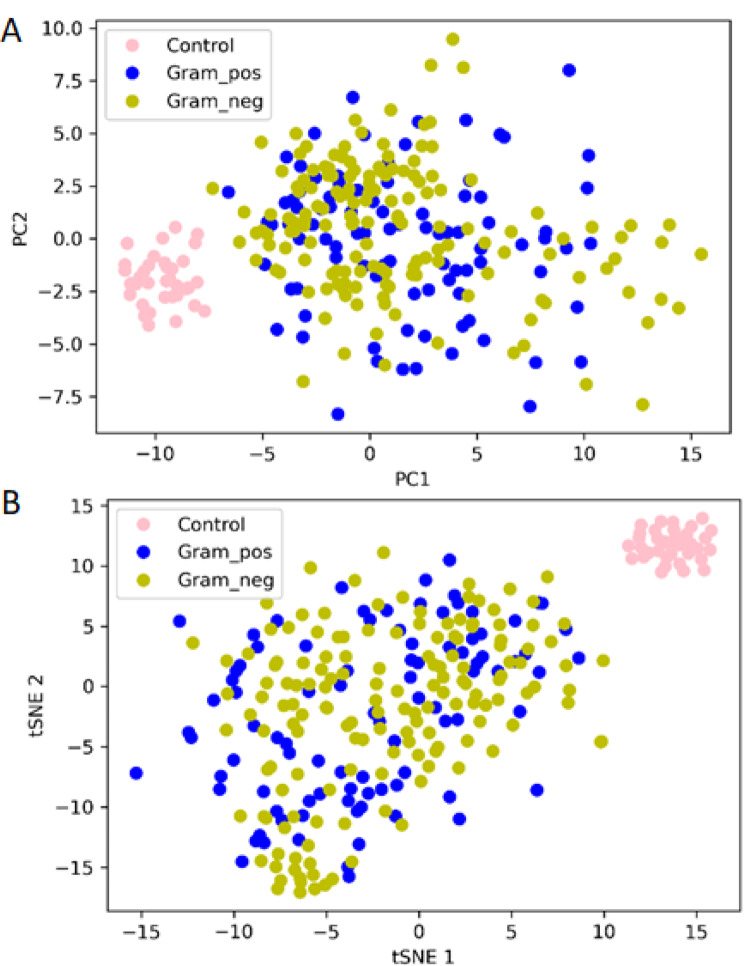
Distribution of samples of patients using the 55 discriminative proteins. **A**) Principal component analysis (PCA) plot **B**) *t*-distributed stochastic neighbor embedding (tSNE) plot. Yellow dots: samples from patients with gram-negative infection, blue dots: samples from patients with gram-positive infection, pink dots: healthy control individuals

To conduct a more detailed evaluation of the 55 discriminative proteins, we replicated unsupervised clustering using the PCA and tSNE plots (Fig. 5). In comparison to the analysis involving all proteins, samples from healthy control individuals formed more distinct clusters. However, no clear separation between samples from patients with gram-negative and gram-positive infections could be observed.

### Detailed analysis of discriminative proteins in the patient cohort

**Fig. 6:**
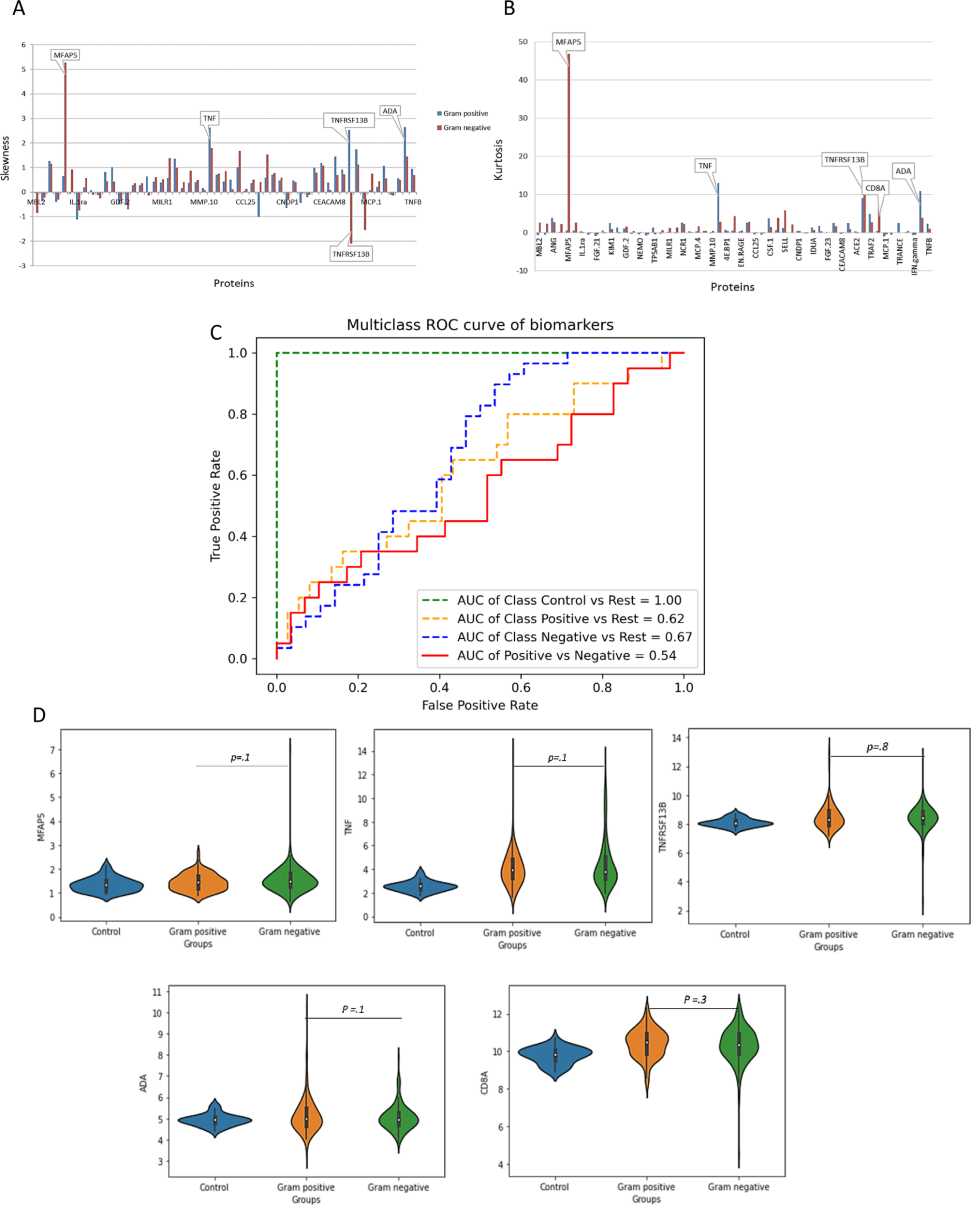
Detailed analysis of selected discriminative proteins. Skewness (**A**) and kurtosis (**B**) of 55 discriminative proteins selected by Lasso (patients with gram-positive infection; blue line, patients with gram-negative infection; red line). (**C**) receiver operating characteristic analysis showing specificity and sensitivity of the set of discriminative proteins after excluding skewed proteins. (**D**) violin plot of five skewed discriminative proteins in patients with gram-negative and gram-positive infection, as assessed by a student t-test with Benjamini-Hochberg adjustment. MFAP5; microfibrillar-associated protein 5, TNF; tumor necrosis factor, TNFRSF13B; TNF receptor superfamily member 13B, ADA; Adenosine Deaminase, CD8A; T-Cell surface glycoprotein CD8 alpha chain

To assess identified proteins in more detail, we determined their distributions using skewness and kurtosis, where diverging values may indicate underlying subpopulations of patients. Skewness and kurtosis were employed for symmetry and peakedness, respectively, with the normal range defined as − 2 to +2 for skewness and −7 to  + 7 for kurtosis [47, 48]. In samples from patients with gram-negative infection, proteins exhibited skewness in the range of +/−2, except for MFAP5 with considerable positive, and TNFRSF13B with negative skewness (Fig. 6A). Kurtosis ranged within +/−7, except for MFAP5, TNFRSF13B, and CD8A proteins, which had higher positive kurtosis (Fig. 6B). Similarly, samples from patients with gram-positive infection, showed skewness within the range, with exceptions for TNF, TNFRSF13B, and ADA having higher positive skewness (Fig. 6A) and limited or positive kurtosis within the range, except for TNF, TNFRSF13B and ADA which had higher positive kurtosis (Fig. 6B).

Further assessment of the impact of MFAP5, TNF, TNFRSF13B, ADA, and CD8A on the performance of the selected proteins in classifying patients with gram-negative and gram-positive infections using healthy controls as reference revealed a reduction in sensitivity and specificity in the absence of these proteins, as evidenced by lower AUC values (AUC 0.67, 95% CI 0.588–0.732 for patients with gram-negative infection from the rest and AUC 0.62, 95% CI 0.549–0.691 for patients with gram-positive infection from the rest) (Fig. 6C). The slight decrease was further supported by student t-test with Benjamini-Hochberg adjustment which revealed no significant differences in the levels of these proteins between patients with gram-negative and gram-positive infections (Fig. 6D). The Violin plot further highlighted high expression pattern similarity, but variations indicated a subpopulation effect in patients’ classification (Additional file 6). This demonstrates that the performance of predictive proteins as a group outperforms their individual performance.

## Discussion

The current study was designed to identify protein biomarkers with potential to discriminate between patients with gram-negative and gram-positive infections using healthy controls as a reference. Utilizing targeted PEA and four panels, we profiled 368 proteins that were potentially related to bacterial sepsis. We applied three different algorithms to identify related proteins (Fig. 1); 1) random forest (RF) which is an ensemble algorithm and offers an adjustment for unbalanced data [42], 2) least absolute shrinkage and selection operator (Lasso), a type of “penalized linear regression” model facilitating automated biomarkers selection [43], and 3) recursive feature elimination (RFE); a wrapper feature selection model allowing to use various machine learning algorithms in the core [49]. The use of the aforementioned algorithm to find predictive biological biomarkers as well as to accurately classify patients has previously been investigated by Chen *et.al* [50], Ghosh and Chinnaiyan [51], and Zhang and Liu [52], respectively.

We applied the three algorithms and evaluated their performance to classify 281 valid samples using ten different inclusion criteria for proteins with missing data, ranging from < 5% to < 80% missing data per protein. The most common approaches for handling missing values below LOD consist of (i) excluding data from analysis [53] that would lead to massive data loss, potential loss of predictive biomarkers, and reduction of statistical power, (ii) replacing missing values with a fixed value [54], which causes variance reduction and subsequently generating bias and (iii) imputation [55] that is preferable for multivariate data analysis. We chose to impute missing values using the GSimp imputation method recommended by Lenz *et.al* for PEA proteomic data [36]. GSimp uses the characteristics of the distribution of the values above LOD to estimate the values below LOD [37]. The performance of the methods was evaluated using metrics of accuracy, precision, recall, f1-score, and MSE. Our data showed that a stringent filtration of proteins with missing values (i.e., < 5%) as well as a non-stringent inclusion criterion (i.e., < 80%), reduced predictive power (Fig. 2). In other words, we found that moderate inclusion criteria of proteins ( < 40% in our case) were optimal, displaying maximum accuracy (Fig. 2). This is in consistence with the manufacturer’s recommended exclusion limit, in the range of less than 25–50% missing value [56]. Furthermore, comparing the accuracies attained in the dataset with 80% missing data compared to the dataset with 5% missing data validates the imputation approach we used. Our top-ranking dataset (i.e., the dataset with < 40% missing value) with 285 proteins (Additional file 3, 4) achieved an accuracy of 63.0% by RF, 71.2% by Lasso, and 67.0% using RFE-LR for classifying patients with gram-negative, and gram-positive infections using healthy control individuals. We used Lasso for the selection of the biomarkers because of the highest accuracy among models. Another benefit of Lasso is its ability to produce a simple and interpretable model. We identified 55 proteins to distinguish between patients with gram-negative and gram-positive infections using healthy controls as reference. We discovered that the prediction performance of the 55 proteins was slightly higher for patients with gram-negative infection than for gram-positive infection. The probability of correctly identifying patients with gram-negative infection from other cases was 69%, while the probability of identifying patients with gram-positive infection correctly was lower at 66% (Fig. 4). Overall, the prediction accuracy was relatively low. One strategy to improve this would be to screen more discriminative proteins, potentially by identifying specific profiles associated with particular bacterial species. However, the limited number of samples per bacterial strain in our dataset hindered the identification of any distinct discriminative proteins. An alternative approach to improve the result could involve conducting a comprehensive analysis of the entire proteome, utilizing techniques such as mass spectrometry. Other studies have presented different results of machine learning methods in differentiating between gram-negative and gram-positive bacterial infections. One study by Zhang *et al*. examined 49 blood biomarkers, encompassing leukocytes, typical cytokines like IFN-γ, chemokines, and molecules linked to tissue damage in peritonitis patients [57]. Using the RFE-RF model, it successfully differentiated gram-negative bacterial infections with an AUC of 0.993, employing a set of eight biomarkers. For the discrimination of gram-positive bacterial infections, the model achieved an AUC of 0.711 with the top five biomarkers [57]. IFN-γ was among the identified biomarkers, and notably, it was also part of the 55 proteins we had identified. In another study using ELISA to examine eight cytokines, patients with gram-negative bacteremia showed increased levels of IFN-γ, TNF-α, IL-1ra, and IL-10 compared to those with gram-positive bacteremia [19]. Notably, these markers were also identified as discriminative proteins in our study, distinguishing between patients with gram-negative and gram-positive infections (Additional file 5).

In this study, we aimed to assess the findings from Lasso feature selection using the distribution and performance of predictive proteins in infection patients. According to Hair *et al*. (2010) and Bryne (2010), normal distribution refers to values of − 2 to +2 for skewness and −7 to + 7 for kurtosis [47, 48]. Based on this recommendation, five proteins (MFAP5, TNF-α, TNFRS13B, ADA, and CD8A) were not normally distributed, which may indicate that underlying subpopulations of patients with gram-negative and gram-positive infections may exist that give rise to changes in protein levels (Fig. 6). However, additional studies are needed to determine the exact relevance of these markers in infection.

An important limitation of this study is the long storage time of the samples prior to analysis, which may have affected the stability and integrity of certain proteins, potentially contributing to the relatively modest classification performance observed. The study population also included both septic and non-septic patients, potentially introducing variability in the observed outcomes. Additionally, the sample sizes were small and uneven across the groups, which may have introduced bias and limited the model’s ability to capture group-specific patterns. As such, the findings should be interpreted within the context of this particular cohort and the targeted PEA method used.

## Conclusion

To the best of our knowledge, our study is one of the first in the field that simultaneously profiled a high number of proteins for the discrimination of gram-negative and gram-positive infections. While 55 proteins demonstrated some discriminative potential, the overall predictive performance was low and did not surpass that of established single biomarkers, limiting its clinical relevance. The findings underscore the challenges of using a multimarker approach for infection classification and highlight the substantial biological overlap between infection types. These results are based on a relatively small and unevenly distributed sample set from a single hospital, including both septic and non-septic patients, and may have been affected by long-term sample storage. Therefore, they may not be generalized to other populations, sample handling conditions, or analytical platforms. Nevertheless, this study provides valuable insight into the limitations of current proteomic strategies for infection diagnostics.

## Electronic supplementary material

Below is the link to the electronic supplementary material.

Supplementary Material 1: Venn diagram of overlap and unique proteins quantified by the four Olink biomarker panels

Supplementary Material 2: Clustered column-line chart representing frequency and fraction of proteins below the LOD value in samples (*n* = 281)

Supplementary Material 3: Summary of ten generated missing thresholds

Supplementary Material 4: Differential expression between the three groups of control, patients with gram-positive, and gram-negative infections after imputation of the top-ranked dataset with 285 proteins

Supplementary Material 5: A ranked list of positive and negative coefficients of discriminative proteins selected by Lasso

Supplementary Material 6: Violen plot of 55 discriminative proteins

## Data Availability

All data is available from the Swedish National Data Service database (https://snd.gu.se/en/catalogue/dataset/preview/fd50cc33-72d9-4185-8435-dbc012b3fcf8/1).

## Abbreviation

AUC: Area under the curve
AUROC: Area under receiver characteristic operator
CM: Cardiometabolic
CVD II: Cardiovascular II
Inf: Inflammation
IR: Immune response
Lasso: Least absolute shrinkage and selection operator
LOD: Limit of Detection
LR: Logistic regression
MNAR: Missing not at random
MS: Mass spectrometry
MSE: Mean square error
PCA: Principal component analysis
PEA: Proximity extension assay
RF: Random forest
RFE: Recursive feature elimination
tSNE: *t*-distributed stochastic neighbour
