# Supplementary material for: Identification of protein biomarkers to differentiate between gram-negative and gram-positive infections in adults suspected of sepsis
Source: BMC Infect Dis. 2025 Nov 14;25:1576. doi: 10.1186/s12879-025-11973-5 (PMC12619434; doi:10.1186/s12879-025-11973-5)
Supplement: Supplementary file 1 — Supplementary Material 1: Venn diagram of overlap and unique proteins quantified by the four Olink biomarker panels [file 12879_2025_11973_MOESM1_ESM.docx]

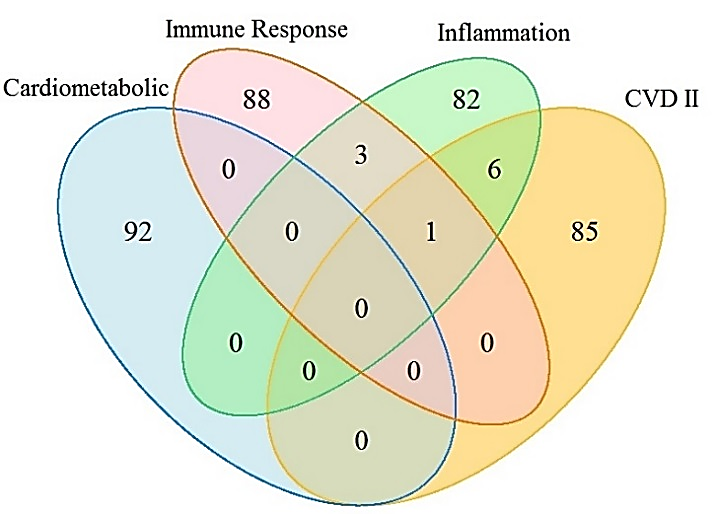


Additional file 1. Venn diagram representing the number of overlap and unique proteins quantified by the four Olink biomarker panels. CVD II, Cardiovascular II.
