# Supplementary material for: Identification of protein biomarkers to differentiate between gram-negative and gram-positive infections in adults suspected of sepsis
Source: BMC Infect Dis. 2025 Nov 14;25:1576. doi: 10.1186/s12879-025-11973-5 (PMC12619434; doi:10.1186/s12879-025-11973-5)
Supplement: Supplementary file 2 — Supplementary Material 2: Clustered column-line chart representing frequency and fraction of proteins below the LOD value in samples (n = 281) [file 12879_2025_11973_MOESM2_ESM.docx]

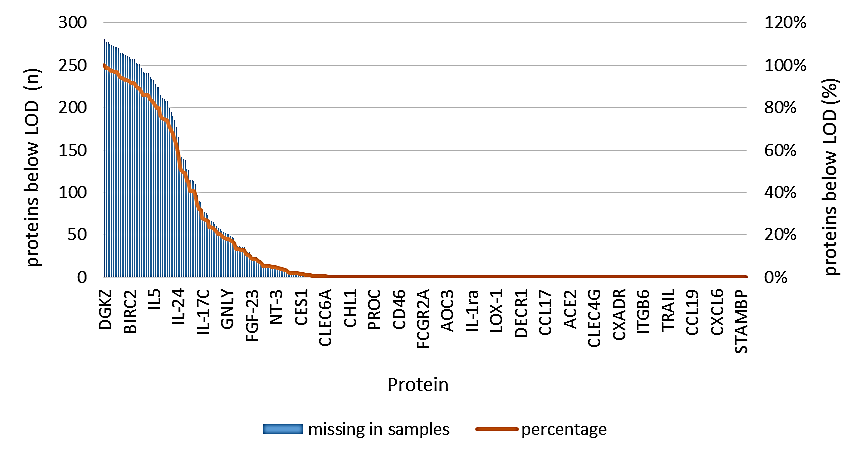


Additional file 2. Clustered column-line chart representing frequency and fraction of proteins below the LOD value in samples (*n* = 281). The names of the proteins appear on the X-axis at intervals of 15 (*n* = 368)
