# Supplementary material for: Identification of protein biomarkers to differentiate between gram-negative and gram-positive infections in adults suspected of sepsis
Source: BMC Infect Dis. 2025 Nov 14;25:1576. doi: 10.1186/s12879-025-11973-5 (PMC12619434; doi:10.1186/s12879-025-11973-5)
Supplement: Supplementary file 3 — Supplementary Material 3: Summary of ten generated missing thresholds [file 12879_2025_11973_MOESM3_ESM.docx]

Additional file 3. Summary of ten generated missing thresholds.

^1^ metrics evaluated using Random Forest and Recursive Feature Elimination-Logistic Regression

^2^ metrics evaluated using Least Absolut Shrinkage and Selection Operator (Lasso).

Gram_neg, gram-negative infection; Gram_pos, gram-positive infection; MSE, Mean Square Error; RF, Random Forest; RFE-LR, Recursive Feature Elimination-Logistic Regression
