# Supplementary material for: Identification of protein biomarkers to differentiate between gram-negative and gram-positive infections in adults suspected of sepsis
Source: BMC Infect Dis. 2025 Nov 14;25:1576. doi: 10.1186/s12879-025-11973-5 (PMC12619434; doi:10.1186/s12879-025-11973-5)
Supplement: Supplementary file 4 — Supplementary Material 4: Differential expression between the three groups of control, patients with gram-positive, and gram-negative infections after imputation of the top-ranked dataset with 285 proteins [file 12879_2025_11973_MOESM4_ESM.docx]

Additional file 4. Differential expression between the three groups of control, patients with gram-positive, and gram-negative infections after imputation of the top-ranked dataset with 285 proteins. The table lists the abbreviation of proteins, mean, standard deviation (std), p-value, the adjusted p-value (Benjamini and Hochberg), and percentage of the missing value of each protein before imputation.

| **Proteins** | **After imputation** | | | | | | | | | **Before imputation** | | |
| --- | --- | --- | --- | --- | --- | --- | --- | --- | --- | --- | --- | --- |
|  | **Control** | **Gram Positive** | **Gram Negative** | **Control/Gram-negative** | | **Control/Gram-positive** | | **Gram-positive/Gram-negative** | | **Missing value (%)** | | |
|  | mean (std) | mean (std) | mean (std) | p-value | p-adj | p-value | p-adj | p-value | p-adj | Control | Gram-positive | Gram-negative |
| CA1 | 4.32 (0.73) | 4.75 (1.58) | 4.63 (1.46) | 4.0002E-100 | 6.1E-100 | 0.125678 | 0.153726261 | 0.556046 | 0.926743814 | 0 | 0 | 0 |
| ICAM1 | 6.04 (0.37) | 6.77 (0.57) | 6.65 (0.64) | 4.3271E-103 | 7E-103 | 1.22E-10 | 6.10453E-10 | 0.127243 | 0.906607872 | 0 | 0 | 0 |
| CHL1 | 3.21 (0.34) | 3.13 (0.44) | 3.1 (0.47) | 3.6482E-132 | 1.9E-131 | 0.332114 | 0.374120415 | 0.645482 | 0.953173519 | 0 | 0 | 0 |
| TGFBI | 8.13 (0.45) | 8.32 (0.54) | 8.15 (0.63) | 2.70226E-63 | 3.16E-63 | 0.066119 | 0.085266556 | 0.028681 | 0.544942947 | 0 | 0 | 0 |
| ENG | 1.8 (0.3) | 1.81 (0.34) | 1.74 (0.36) | 1.0444E-144 | 1.6E-143 | 0.922866 | 0.946103375 | 0.148558 | 0.882063991 | 0 | 0 | 0 |
| PLTP | 1.84(0.28) | 1.81 (0.39) | 1.79 (0.44) | 3.1651E-145 | 5.3E-144 | 0.703933 | 0.737576629 | 0.689147 | 0.958082282 | 2.86 | 8.70 | 7.14 |
| SERPINA7 | 4.61 (0.4) | 4.35 (0.55) | 4.39 (0.46) | 7.7751E-117 | 1.8E-116 | 0.010952 | 0.015844736 | 0.555888 | 0.931930621 | 0 | 0 | 0 |
| IGFBP3 | 3.57 (0.41) | 3.23 (0.69) | 3.2 (0.78) | 4.5506E-125 | 1.5E-124 | 0.007703 | 0.011554878 | 0.771937 | 0.982151982 | 0 | 0 | 0 |
| CR2 | 6.4 (0.63) | 5.96 (0.85) | 5.84 (0.93) | 6.33868E-83 | 8.4E-83 | 0.006657 | 0.010091954 | 0.300574 | 0.815844744 | 0 | 0 | 0 |
| SERPINA5 | 7.84 (0.49) | 6.55 (0.9) | 6.55 (0.9) | 2.5906E-67 | 3.1E-67 | 5.82E-13 | 3.60711E-12 | 0.991983 | 0.998993712 | 0 | 0 | 0 |
| FCGR3B | 3.51 (0.57) | 3.84 (0.74) | 3.74 (0.69) | 1.2393E-115 | 2.8E-115 | 0.020875 | 0.029020945 | 0.279288 | 0.804010224 | 0 | 0 | 0 |
| IGFBP6 | 5.57 (0.41) | 5.78 (0.67) | 5.88 (0.89) | 2.5562E-106 | 4.6E-106 | 0.079104 | 0.101096515 | 0.334015 | 0.827777384 | 0 | 0 | 0 |
| CDH1 | 2.98 (0.34) | 3.15 (0.6) | 3.2 (0.73) | 3.8489E-134 | 2.1E-133 | 0.123097 | 0.151873159 | 0.601904 | 0.963722598 | 0 | 0 | 0 |
| CCL5 | 3.86 (0.76) | 3.87 (1.04) | 3.88 (0.99) | 1.7181E-102 | 2.8E-102 | 0.957542 | 0.964309156 | 0.942387 | 1 | 0 | 0 | 0 |
| CCL14 | 5.7 (0.41) | 6.51 (0.73) | 6.52 (0.77) | 1.2868E-104 | 2.2E-104 | 7.32E-09 | 2.60936E-08 | 0.901297 | 1 | 0 | 0 | 0 |
| NOTCH1 | 3.13 (0.38) | 3.13 (0.35) | 3.08 (0.37) | 3.3146E-130 | 1.4E-129 | 0.954745 | 0.964901486 | 0.313237 | 0.826598685 | 0 | 0 | 0 |
| PAM | 1.52 (0.36) | 1.59 (0.37) | 1.51 (0.46) | 1.4585E-142 | 1.6E-141 | 0.312419 | 0.354738331 | 0.154964 | 0.88329268 | 0 | 0 | 0 |
| PROC | 4.24 (0.39) | 3.8 (0.51) | 3.81 (0.65) | 5.8927E-121 | 1.6E-120 | 1.07E-05 | 2.31074E-05 | 0.905802 | 1 | 0 | 0 | 0 |
| CST3 | 5.81 (0.49) | 6.78 (0.81) | 6.82 (0.87) | 1.32519E-98 | 1.98E-98 | 8.74E-10 | 3.60849E-09 | 0.737382 | 0.986637355 | 0 | 0 | 0 |
| NCAM1 | 3.55 (0.35) | 3.28 (0.41) | 3.31 (0.42) | 7.4065E-129 | 2.7E-128 | 0.000889 | 0.001544319 | 0.621391 | 0.957277501 | 0 | 0 | 0 |
| PCOLCE | 5.57 (0.44) | 5.26 (0.5) | 5.25 (0.72) | 2.3803E-104 | 4E-104 | 0.001559 | 0.002629895 | 0.936242 | 1 | 0 | 0 | 0 |
| LILRB1 | 2.25 (0.47) | 2.78 (0.7) | 2.78 (0.61) | 2.6631E-131 | 1.2E-130 | 5.21E-05 | 0.000103095 | 0.986868 | 1 | 0 | 0 | 0 |
| MET | 1.97 (0.29) | 2.08 (0.34) | 2.06 (0.38) | 2.8478E-144 | 3.7E-143 | 0.099208 | 0.12455575 | 0.612325 | 0.948438331 | 0 | 0 | 0 |
| IL7R | 1.84 (0.38) | 1.83 (0.46) | 1.65 (0.47) | 1.912E-139 | 1.6E-138 | 0.934888 | 0.951582741 | 0.002998 | 0.427168682 | 5.71 | 9.78 | 26.62 |
| VCAM1 | 4.12 (0.46) | 4.86 (0.6) | 4.87 (0.65) | 1.474E-117 | 3.5E-117 | 1.08E-09 | 4.32594E-09 | 0.916445 | 1 | 0 | 0 | 0 |
| SELL | 8.24 (0.44) | 8.24 (0.54) | 8.06 (0.6) | 5.59885E-61 | 6.41E-61 | 0.941003 | 0.95439823 | 0.018019 | 0.427953576 | 0 | 0 | 0 |
| F11 | 6.64 (0.39) | 6.32 (0.56) | 6.36 (0.57) | 5.83506E-94 | 8.31E-94 | 0.002657 | 0.004253936 | 0.562892 | 0.927308121 | 0 | 0 | 0 |
| COMP | 7.51 (0.38) | 7.11 (0.64) | 6.95 (0.78) | 4.82742E-80 | 6.28E-80 | 0.000602 | 0.001079745 | 0.088809 | 0.816472248 | 0 | 0 | 0 |
| CA4 | 1.38 (0.29) | 1.6 (0.44) | 1.56 (0.51) | 8.1693E-148 | 1.9E-146 | 0.006643 | 0.010123955 | 0.531897 | 0.924333241 | 5.71 | 3.26 | 6.49 |
| PTPRS | 0.65 (0.31) | 0.56 (0.29) | 0.53 (0.3) | 4.1406E-151 | 1.7E-149 | 0.131858 | 0.160596155 | 0.496365 | 0.936848071 | 0 | 0 | 0 |
| MBL2 | 8.72 (0.77) | 8.84 (1.08) | 8.9 (1.2) | 2.38455E-33 | 2.54E-33 | 0.561627 | 0.601742742 | 0.685663 | 0.957911031 | 0 | 0 | 0 |
| TIMP1 | 4.14 (0.41) | 5.71 (1.1) | 5.6 (0.94) | 1.0714E-120 | 2.9E-120 | 2.1E-13 | 1.38953E-12 | 0.393064 | 0.855140164 | 0 | 0 | 0 |
| ANGPTL3 | 5.49 (0.41) | 5.53 (0.62) | 5.6 (0.67) | 7.235E-107 | 1.3E-106 | 0.726147 | 0.758065221 | 0.374361 | 0.84676828 | 0 | 0 | 0 |
| CD46 | 3.17 (0.36) | 3.38 (0.52) | 3.43 (0.61) | 9.9639E-132 | 4.8E-131 | 0.030468 | 0.041153722 | 0.483278 | 0.936967028 | 0 | 0 | 0 |
| TNC | 3.65 (0.61) | 5.11 (0.99) | 5.04 (1.01) | 2.472E-112 | 5E-112 | 3.82E-13 | 2.47647E-12 | 0.604987 | 0.963247667 | 0 | 0 | 0 |
| NID1 | 3.53 (0.4) | 4.48 (0.59) | 4.41 (0.65) | 4.0054E-126 | 1.4E-125 | 1.17E-14 | 9.28862E-14 | 0.39676 | 0.856641839 | 0 | 0 | 0 |
| CFHR5 | 7.46 (0.45) | 8.01 (0.72) | 8.03 (0.78) | 1.24385E-76 | 1.58E-76 | 4.8E-05 | 9.56155E-05 | 0.819653 | 0.998295531 | 0 | 0 | 0 |
| SPARCL1 | 3.06 (0.41) | 3.14 (0.42) | 3.2 (0.53) | 3.0263E-129 | 1.2E-128 | 0.30952 | 0.355698868 | 0.364609 | 0.858790275 | 0 | 0 | 0 |
| PLXNB2 | 1.39 (0.28) | 1.66 (0.33) | 1.66 (0.38) | 4.6527E-148 | 1.3E-146 | 3.22E-05 | 6.5597E-05 | 0.896799 | 1 | 0 | 1.09 | 0.65 |
| MEGF9 | 3.66 (0.32) | 3.56 (0.42) | 3.53 (0.41) | 3.4086E-130 | 1.4E-129 | 0.162435 | 0.194512951 | 0.635259 | 0.952888814 | 0 | 0 | 0 |
| ANG | 6.3 (0.38) | 6.43 (0.77) | 6.54 (0.76) | 2.897E-99 | 4.3E-99 | 0.338034 | 0.377802499 | 0.256439 | 0.812055859 | 0 | 0 | 0 |
| ST6GAL1 | 2.82 | 3.93 (0.69) | 3.89 (0.72) | 7.3099E-135 | 4.3E-134 | 3.21E-15 | 2.68939E-14 | 0.65216 | 0.948293926 | 0 | 0 | 0 |
| DPP4 | 4.5 (0.37) | 4.14 (0.52) | 4.01 (0.49) | 6.0158E-120 | 1.6E-119 | 0.000255 | 0.000478434 | 0.048356 | 0.574233024 | 0 | 0 | 0 |
| REG1A | 6.16 (0.54) | 7.88 (1.77) | 7.74 (1.5) | 5.13382E-91 | 7.21E-91 | 1.06E-07 | 3.38756E-07 | 0.505118 | 0.934795631 | 0 | 0 | 0 |
| FCN2 | 5.36 (0.47) | 5.77 (0.73) | 5.71 (0.7) | 1.2975E-104 | 2.2E-104 | 0.00232 | 0.003822416 | 0.546704 | 0.927444201 | 0 | 0 | 0 |
| FETUB | 2.08 (0.43) | 1.66 (0.56) | 1.59 (0.64) | 1.2773E-134 | 7.3E-134 | 0.000121 | 0.000233758 | 0.364612 | 0.851757082 | 0 | 0 | 0.65 |
| CES1 | 2.79 (0.69) | 3.56 (1.07) | 3.57 (1.31) | 1.8883E-114 | 4E-114 | 0.000122 | 0.000233654 | 0.972195 | 1 | 2.86 | 1.09 | 1.30 |
| CRTAC1 | 3.5 (0.54) | 2.74 (0.61) | 2.72 (0.68) | 1.1312E-117 | 2.7E-117 | 1.63E-09 | 6.35131E-09 | 0.815612 | 0.997636977 | 0 | 0 | 0 |
| TCN2 | 4.28 (0.38) | 4.7 (0.69) | 4.66 (0.61) | 3.5133E-121 | 9.7E-121 | 0.000866 | 0.001513681 | 0.652559 | 0.944056822 | 0 | 0 | 0 |
| PRSS2 | 2.63 (0.45) | 3.04 (0.97) | 3.26 (1.36) | 5.983E-130 | 2.4E-129 | 0.018963 | 0.026491904 | 0.179127 | 0.82340674 | 0 | 0 | 1.30 |
| ICAM3 | 2.67 (0.33) | 2.85 (0.48) | 2.78 (0.52) | 3.5936E-137 | 2.6E-136 | 0.049082 | 0.064462655 | 0.272386 | 0.800310389 | 0 | 0 | 0.65 |
| SAA4 | 3.97 (0.65) | 4.18 (0.8) | 4.15 (0.76) | 2.3429E-107 | 4.3E-107 | 0.154112 | 0.186109908 | 0.754323 | 0.981653498 | 0 | 0 | 0 |
| CNDP1 | 5.03 (0.52) | 3.66 (0.84) | 3.59 (0.83) | 3.1955E-105 | 5.6E-105 | 4.77E-15 | 3.88131E-14 | 0.524889 | 0.929151556 | 0 | 1.09 | 0 |
| FCGR2A | 3.03 (0.72) | 3.51 (0.75) | 3.51 (0.67) | 6.1942E-111 | 1.2E-110 | 0.001407 | 0.002415889 | 0.95649 | 1 | 0 | 0 | 0 |
| NRP1 | 1.07 (0.21) | 1.23 (0.27) | 1.23 (0.38) | 7.9322E-154 | 5.7E-152 | 0.002122 | 0.003515463 | 0.999052 | 0.999052373 | 0 | 0 | 0 |
| EFEMP1 | 5.44 (0.42) | 5.94 (0.64) | 5.99 (0.78) | 7.1004E-107 | 1.3E-106 | 4.19E-05 | 8.40719E-05 | 0.608302 | 0.963144673 | 0 | 0 | 0 |
| TIMD4 | 4.09 (0.47) | 5.02 (0.83) | 4.95 (0.9) | 3.4062E-117 | 8E-117 | 6.39E-09 | 2.33596E-08 | 0.532631 | 0.919998365 | 0 | 0 | 0 |
| TIE1 | 1.61 (0.32) | 1.8 (0.32) | 1.78 (0.42) | 1.3164E-144 | 1.8E-143 | 0.005472 | 0.008476419 | 0.787699 | 0.984623807 | 0 | 0 | 0 |
| THBS4 | 4.58 (0.5) | 4.66 (0.8) | 4.53 (0.92) | 4.8458E-111 | 9.6E-111 | 0.561594 | 0.603978128 | 0.25406 | 0.813562289 | 0 | 0 | 0 |
| F7 | 3.56 (0.35) | 3.37 (0.47) | 3.38 (0.63) | 5.5428E-129 | 2.1E-128 | 0.030874 | 0.041505198 | 0.912892 | 1 | 0 | 0 | 1.95 |
| GP1BA | 5.16 (0.41) | 5.17 (0.49) | 5.25 (0.58) | 8.8546E-111 | 1.7E-110 | 0.927155 | 0.947093809 | 0.262831 | 0.814205205 | 0 | 0 | 0 |
| LYVE1 | 5.52 (0.39) | 5.95 (0.6) | 6 (0.71) | 7.0438E-108 | 1.3E-107 | 0.000132 | 0.000250372 | 0.536279 | 0.920720815 | 0 | 0 | 0 |
| CA3 | 1 (0.38) | 2.28 (1.63) | 2.24 (1.37) | 7.986E-145 | 1.3E-143 | 1.19E-05 | 2.54275E-05 | 0.830593 | 1 | 2.86 | 1.09 | 2.60 |
| TGFBR3 | 3.21 (0.36) | 3.36 (0.48) | 3.37 (0.64) | 5.9379E-131 | 2.6E-130 | 0.093653 | 0.118627721 | 0.899656 | 1 | 0 | 0 | 0 |
| APOM | 6.01 (0.42) | 5.31 (0.63) | 5.32 (0.6) | 4.2916E-100 | 6.5E-100 | 1.78E-08 | 6.20155E-08 | 0.938843 | 1 | 0 | 0 | 0 |
| OSMR | 0.69 (0.21) | 0.93 (0.27) | 0.91 (0.28) | 2.439E-156 | 3.5E-154 | 4.68E-06 | 1.12923E-05 | 0.702603 | 0.962701275 | 2.86 | 0 | 0 |
| LILRB2 | 3.26 (0.37) | 3.84 (0.64) | 3.84 (0.7) | 4.2509E-130 | 1.7E-129 | 1.14E-06 | 3.02886E-06 | 0.958211 | 1 | 0 | 0 | 0 |
| CCL18 | 6.07 (0.58) | 7.28 (1.16) | 7.26 (1.14) | 5.21704E-90 | 7.22E-90 | 3.83E-08 | 1.25483E-07 | 0.919701 | 1 | 0 | 0 | 0 |
| COL18A1 | 2.99 (0.33) | 3.74 (0.72) | 3.78 (0.75) | 1.7927E-134 | 1E-133 | 2.1E-08 | 7.22366E-08 | 0.675831 | 0.963059689 | 0 | 0 | 0 |
| KIT | 4.04 (0.46) | 3.48 (0.55) | 3.39 (0.54) | 6.0739E-118 | 1.5E-117 | 4.14E-07 | 1.18106E-06 | 0.213349 | 0.832938937 | 0 | 0 | 1.30 |
| C1QTNF1 | 4.48 (0.41) | 5.45 (0.8) | 5.33 (0.77) | 4.3956E-117 | 1E-116 | 4.35E-10 | 1.87847E-09 | 0.235386 | 0.808253209 | 0 | 0 | 0 |
| AOC3 | 3.24 (0.34) | 3.17 (0.52) | 3.16 (0.46) | 6.0896E-132 | 3E-131 | 0.500893 | 0.54073672 | 0.813463 | 0.99929692 | 0 | 0 | 0 |
| GAS6 | 4.37 (0.39) | 5.05 (0.45) | 5.06 (0.65) | 1.4221E-119 | 3.8E-119 | 2.14E-12 | 1.24725E-11 | 0.937446 | 1 | 0 | 0 | 0 |
| IGLC2 | 5.52 (0.56) | 6.31 (0.91) | 6.23 (0.99) | 1.38008E-97 | 2.04E-97 | 3.48E-06 | 8.61997E-06 | 0.489848 | 0.930710853 | 0 | 0 | 0 |
| TNXB | 1.23 (0.18) | 1.13 (0.27) | 1.06 (0.28) | 2.446E-154 | 2.3E-152 | 0.038297 | 0.050765385 | 0.078742 | 0.748045152 | 0 | 10.87 | 9.09 |
| MFAP5 | 1.36 (0.3) | 1.46 (0.36) | 1.57 (0.59) | 1.827E-147 | 4E-146 | 0.165248 | 0.197053485 | 0.112175 | 0.819742479 | 2.86 | 3.26 | 4.55 |
| VASN | 1.62 (0.26) | 1.65 (0.3) | 1.68 (0.36) | 5.9888E-148 | 1.6E-146 | 0.661315 | 0.703264419 | 0.478255 | 0.946547166 | 0 | 0 | 0 |
| LILRB5 | 4.61 (0.68) | 4.18 (0.83) | 4.21 (0.87) | 3.6877E-100 | 5.7E-100 | 0.006992 | 0.010544169 | 0.744081 | 0.9772493 | 0 | 0 | 0 |
| C2 | 6.2 (0.39) | 6.44 (0.57) | 6.46 (0.5) | 9.1705E-100 | 1.4E-99 | 0.023639 | 0.032546041 | 0.780636 | 0.984429768 | 0 | 0 | 0 |
| BMP.6 | 4.45 (0.27) | 4.87 (0.48) | 4.93 (0.43) | 9.6287E-126 | 3.2E-125 | 4.12E-06 | 1.00453E-05 | 0.271567 | 0.814701886 | 0 | 0 | 0 |
| ANGPT1 | 6.64 (0.53) | 6.85 (0.72) | 6.98 (0.71) | 3.4808E-85 | 4.75E-85 | 0.110022 | 0.137527304 | 0.165549 | 0.890215949 | 0 | 0 | 0 |
| ADM | 6.79 (0.32) | 8.41 (0.66) | 8.39 (0.76) | 1.6135E-95 | 2.33E-95 | 6.4E-27 | 2.60442E-25 | 0.835893 | 1 | 0 | 0 | 0 |
| CD40.L | 4.22 (0.68) | 4.34 (0.92) | 4.44 (0.95) | 6.2536E-104 | 1E-103 | 0.476066 | 0.519841937 | 0.424078 | 0.875812473 | 0 | 0 | 0 |
| PGF | 7.75 (0.33) | 8.94 (0.67) | 8.79 (0.71) | 2.2411E-78 | 2.86E-78 | 9.17E-18 | 1.04519E-16 | 0.109204 | 0.819026511 | 0 | 0 | 0 |
| ADAM.TS13 | 6.67 (0.16) | 6.39 (0.26) | 6.41 (0.26) | 2.2923E-105 | 4.1E-105 | 3.61E-08 | 1.19643E-07 | 0.579554 | 0.943845561 | 0 | 0 | 0 |
| BOC | 3.73 (0.28) | 3.44 (0.45) | 3.44 (0.46) | 1.5787E-131 | 7.5E-131 | 0.000709 | 0.001262932 | 0.947508 | 1 | 0 | 0 | 0 |
| IL.4RA | 1.8 (0.28) | 3.15 (0.98) | 3.17 (1.03) | 6.0024E-146 | 1.1E-144 | 6.47E-13 | 3.92239E-12 | 0.898342 | 1 | 31.43 | 0 | 2.60 |
| SRC | 6.57 (0.78) | 6.63 (0.8) | 6.75 (0.74) | 4.75562E-73 | 5.84E-73 | 0.680432 | 0.715583722 | 0.224975 | 0.82202516 | 0 | 0 | 0 |
| IL.1ra | 4.42 (0.66) | 7.35 (0.84) | 7.24 (0.84) | 3.9139E-103 | 6.4E-103 | 9.94E-38 | 2.83374E-35 | 0.29746 | 0.815155702 | 0 | 0 | 0 |
| IL6 | 2.81 (0.49) | 9.89 (2.45) | 9.11 (2.38) | 3.3389E-126 | 1.1E-125 | 4.11E-34 | 5.85122E-32 | 0.014951 | 0.608709768 | 0 | 0 | 0 |
| TNFRSF10A | 2.67 (0.4) | 4.19 (0.78) | 4.21 (0.76) | 1.0869E-132 | 5.6E-132 | 5.22E-20 | 8.26379E-19 | 0.880221 | 1 | 0 | 0 | 0 |
| STK4 | 2.83 (0.88) | 3.69 (0.86) | 3.82 (0.82) | 1.4878E-104 | 2.5E-104 | 1.72E-06 | 4.54382E-06 | 0.263788 | 0.799782913 | 5.71 | 0 | 0 |
| IDUA | 4.87 (0.47) | 4.7 (0.69) | 4.7 (0.71) | 3.6798E-110 | 7.1E-110 | 0.186061 | 0.220031186 | 0.939678 | 1 | 0 | 0 | 0 |
| TNFRSF11A | 5.05 (0.42) | 6.48 (1.1) | 6.65 (1.18) | 4.1945E-111 | 8.4E-111 | 1.19E-11 | 6.53119E-11 | 0.272167 | 0.807995353 | 0 | 0 | 0 |
| PAR.1 | 7.56 (0.38) | 8.13 (0.55) | 8.19 (0.56) | 2.87334E-79 | 3.69E-79 | 1.1E-07 | 3.4744E-07 | 0.407111 | 0.865871092 | 0 | 0 | 0 |
| TRAIL.R2 | 5.06 (0.45) | 6.95 (1.05) | 6.98 (1.11) | 2.869E-109 | 5.5E-109 | 2.21E-18 | 2.62752E-17 | 0.837127 | 1 | 0 | 0 | 0 |
| PRSS27 | 8.59 (0.61) | 8.24 (0.57) | 8.4 (0.57) | 1.11738E-43 | 1.22E-43 | 0.002724 | 0.004337302 | 0.037333 | 0.56000167 | 0 | 0 | 0 |
| TIE2 | 7.15 (0.23) | 7.24 (0.29) | 7.24 (0.32) | 1.05745E-94 | 1.52E-94 | 0.097026 | 0.122355816 | 0.980189 | 1 | 0 | 0 | 0 |
| TF | 5.08 (0.29) | 5.15 (0.49) | 5.24 (0.53) | 1.8304E-118 | 4.5E-118 | 0.415485 | 0.455435352 | 0.172896 | 0.849576303 | 0 | 0 | 0 |
| IL1RL2 | 4.37 (0.43) | 4.68 (0.63) | 4.8 (0.77) | 6.0982E-117 | 1.4E-116 | 0.009977 | 0.014507865 | 0.204016 | 0.842676429 | 0 | 0 | 0 |
| PDGF.subunit.B | 8.59 (0.64) | 8.8 (0.96) | 8.93 (0.92) | 2.62747E-42 | 2.84E-42 | 0.22528 | 0.264217429 | 0.294516 | 0.822912082 | 0 | 0 | 0 |
| IL.27 | 6.4 (0.38) | 7.77 (0.66) | 7.8 (0.75) | 1.63068E-97 | 2.4E-97 | 1.57E-21 | 2.80181E-20 | 0.742244 | 0.979349156 | 0 | 0 | 0 |
| IL.17D | 2.63 (0.66) | 2.73 (0.49) | 2.86 (0.46) | 3.9523E-117 | 9.2E-117 | 0.370039 | 0.411957779 | 0.030115 | 0.476818179 | 0 | 3.26 | 1.30 |
| CXCL1 | 7.95 (1.11) | 8.93 (1.08) | 9.04 (1.33) | 6.44762E-38 | 6.88E-38 | 1.31E-05 | 2.77676E-05 | 0.511957 | 0.923467126 | 0 | 0 | 0 |
| LOX.1 | 5.68 (0.47) | 7.59 (0.93) | 7.4 (1) | 4.1375E-101 | 6.4E-101 | 1.32E-21 | 2.50702E-20 | 0.151291 | 0.879956311 | 0 | 0 | 0 |
| Gal.9 | 7.57 (0.42) | 8.16 (0.59) | 8.26 (0.53) | 1.30832E-76 | 1.65E-76 | 2.73E-07 | 8.11554E-07 | 0.188187 | 0.800495978 | 0 | 0 | 0 |
| GIF | 7.63 (0.87) | 6.65 (1.52) | 6.88 (1.44) | 2.11142E-52 | 2.36E-52 | 0.000493 | 0.000906191 | 0.237901 | 0.807162735 | 0 | 1.09 | 0 |
| SCF | 8.92 (0.36) | 8.19 (0.9) | 8.34 (0.84) | 6.6709E-46 | 7.28E-46 | 7.91E-06 | 1.83324E-05 | 0.18292 | 0.81456563 | 0 | 0 | 0 |
| IL18 | 7.54 (0.55) | 8.34 (0.97) | 8.27 (0.76) | 2.09726E-69 | 2.53E-69 | 8.32E-06 | 1.89627E-05 | 0.524924 | 0.923476965 | 0 | 0 | 0 |
| FGF.21 | 4.77 (1.4) | 8.49 (2.24) | 8.77 (2.28) | 6.54049E-68 | 7.87E-68 | 1.5E-15 | 1.29366E-14 | 0.341278 | 0.831317922 | 0 | 0 | 0 |
| PIgR | 2.3 (0.2) | 2.35 (0.26) | 2.42 (0.36) | 1.3371E-146 | 2.7E-145 | 0.240485 | 0.279748261 | 0.096819 | 0.811572074 | 14.29 | 8.70 | 11.04 |
| RAGE | 12.31 (0.5) | 12.45 (0.69) | 12.51 (0.67) | 4.13767E-59 | 4.7E-59 | 0.279073 | 0.322007577 | 0.504072 | 0.938957691 | 0 | 0 | 0 |
| SOD2 | 9.01 (0.11) | 9.01 (0.11) | 9 (0.14) | 2.69959E-53 | 3.03E-53 | 0.859465 | 0.887491462 | 0.894994 | 1 | 0 | 0 | 0 |
| CTRC | 10.34 (0.55) | 8.53 (1.22) | 8.81 (1.53) | 0.211343093 | 0.211343 | 9.04E-14 | 6.28463E-13 | 0.145646 | 0.883173864 | 0 | 0 | 0 |
| FGF.23 | 2.9 (0.51) | 4.81 (2.06) | 4.54 (2.03) | 4.9526E-124 | 1.6E-123 | 3.17E-07 | 9.22916E-07 | 0.321953 | 0.82663605 | 11.43 | 9.78 | 8.44 |
| SPON2 | 8.94 (0.23) | 9.19 (0.27) | 9.23 (0.24) | 1.02605E-51 | 1.14E-51 | 6.5E-06 | 1.54319E-05 | 0.140854 | 0.933569281 | 0 | 0 | 0 |
| GH | 6.07 (2.04) | 9.12 (1.3) | 8.57 (1.71) | 2.157E-39 | 2.32E-39 | 1.24E-17 | 1.35901E-16 | 0.008406 | 0.59891218 | 0 | 0 | 0 |
| FS | 11 (0.36) | 11.05 (0.82) | 11.18 (0.7) | 1.07455E-24 | 1.11E-24 | 0.73953 | 0.769218663 | 0.168503 | 0.873151655 | 0 | 0 | 0 |
| GLO1 | 6.69 (0.58) | 6.94 (0.79) | 6.97 (0.76) | 5.71471E-82 | 7.54E-82 | 0.08644 | 0.109979434 | 0.794226 | 0.984150213 | 0 | 0 | 0 |
| CD84 | 4.29 (0.34) | 4.53 (0.44) | 4.59 (0.46) | 1.0649E-123 | 3.3E-123 | 0.003775 | 0.005976389 | 0.314063 | 0.821172643 | 0 | 0 | 0 |
| PAPPA | 3.19 (0.5) | 3.56 (0.93) | 3.6 (0.91) | 1.6394E-122 | 4.7E-122 | 0.028485 | 0.038843676 | 0.741469 | 0.987469916 | 14.29 | 8.70 | 11.04 |
| SERPINA12 | 2.72 (1.16) | 2.13 (1.22) | 2.18 (1.08) | 1.22164E-92 | 1.73E-92 | 0.014356 | 0.020560241 | 0.751522 | 0.982494463 | 5.71 | 15.22 | 12.99 |
| REN | 5.85 (0.74) | 6.98 (0.98) | 7.07 (1) | 4.04301E-84 | 5.46E-84 | 8.76E-09 | 3.08256E-08 | 0.479642 | 0.942744015 | 0 | 0 | 0 |
| DECR1 | 6.67 (0.75) | 7.05 (1.09) | 7.28 (1.06) | 4.59376E-73 | 5.67E-73 | 0.060686 | 0.078975226 | 0.099108 | 0.784601524 | 0 | 0 | 0 |
| MERTK | 5.4 (0.34) | 5.71 (0.55) | 5.84 (0.55) | 2.1649E-112 | 4.4E-112 | 0.002402 | 0.003933727 | 0.065152 | 0.687712925 | 0 | 0 | 0 |
| KIM1 | 7.45 (0.64) | 8.96 (1.15) | 9.28 (1.11) | 3.81522E-66 | 4.49E-66 | 2.87E-11 | 1.54173E-10 | 0.028964 | 0.515928024 | 0 | 0 | 0 |
| THBS2 | 5.44 (0.21) | 5.57 (0.3) | 5.65 (0.29) | 5.6715E-119 | 1.4E-118 | 0.016643 | 0.023481478 | 0.029798 | 0.499558482 | 0 | 0 | 0 |
| TM | 9.61 (0.36) | 10.01 (0.52) | 10.02 (0.6) | 1.38298E-19 | 1.41E-19 | 5.54E-05 | 0.000108825 | 0.92258 | 1 | 0 | 0 | 0 |
| VSIG2 | 3.61 (0.4) | 4.34 (0.88) | 4.64 (0.97) | 9.4414E-126 | 3.1E-125 | 7.18E-06 | 1.67633E-05 | 0.01688 | 0.481089345 | 0 | 0 | 0 |
| AMBP | 7.31 (0.19) | 7.66 (0.28) | 7.7 (0.35) | 5.40495E-94 | 7.74E-94 | 1.98E-10 | 9.42309E-10 | 0.387518 | 0.862833803 | 0 | 0 | 0 |
| PRELP | 7.47 (0.2) | 7.78 (0.33) | 7.83 (0.32) | 1.33164E-90 | 1.86E-90 | 4.11E-07 | 1.18405E-06 | 0.30235 | 0.805323828 | 0 | 0 | 0 |
| HO.1 | 11.18 (0.37) | 11.65 (0.53) | 11.66 (0.58) | 5.73126E-32 | 6.05E-32 | 4.03E-06 | 9.89576E-06 | 0.917342 | 1 | 0 | 0 | 0 |
| XCL1 | 4.61 (0.47) | 5.34 (0.82) | 5.28 (0.88) | 1.0623E-112 | 2.2E-112 | 2.22E-06 | 5.70439E-06 | 0.592746 | 0.954421983 | 0 | 0 | 0 |
| IL16 | 5.74 (0.41) | 6.5 (0.76) | 6.47 (0.76) | 2.602E-104 | 4.4E-104 | 1.39E-07 | 4.34247E-07 | 0.769732 | 0.98373872 | 0 | 0 | 0 |
| SORT1 | 7.82 (0.28) | 7.97 (0.36) | 8.09 (0.35) | 1.83709E-79 | 2.37E-79 | 0.031159 | 0.041691616 | 0.010917 | 0.622275128 | 0 | 0 | 0 |
| CEACAM8 | 3.43 (0.57) | 5.59 (1.13) | 5.38 (1.1) | 1.2631E-116 | 2.9E-116 | 1.23E-19 | 1.8445E-18 | 0.14226 | 0.921459931 | 0 | 0 | 0.65 |
| PTX3 | 3.53 (0.44) | 5.52 (0.73) | 5.48 (0.75) | 1.5365E-123 | 4.6E-123 | 8.51E-30 | 4.85036E-28 | 0.650912 | 0.951333364 | 0 | 0 | 0 |
| PSGL.1 | 4.13 (0.32) | 3.92 (0.28) | 3.88 (0.43) | 7.9598E-126 | 2.7E-125 | 0.000448 | 0.000829441 | 0.445967 | 0.90786214 | 0 | 0 | 0 |
| CCL17 | 6.95 (0.49) | 7.16 (1.32) | 7.03 (0.98) | 3.82375E-83 | 5.12E-83 | 0.37127 | 0.411720191 | 0.401198 | 0.859709125 | 0 | 0 | 0 |
| CCL3 | 5.2 (0.5) | 6.94 (1.5) | 7.14 (1.93) | 4.1714E-105 | 7.2E-105 | 5.58E-10 | 2.34014E-09 | 0.407377 | 0.860018568 | 0 | 0 | 0 |
| MMP7 | 10.47 (0.39) | 10.86 (0.44) | 11.01 (0.44) | 0.000286766 | 0.000288 | 9.23E-06 | 2.03843E-05 | 0.015343 | 0.546583042 | 0 | 0 | 0 |
| IgG.Fc.receptor.II.b | 3.65 (0.74) | 3.82 (0.81) | 3.96 (0.95) | 3.5182E-105 | 6.2E-105 | 0.264692 | 0.306654976 | 0.246265 | 0.797564286 | 2.86 | 2.17 | 1.95 |
| DCN | 4.1 (0.24) | 4.58 (0.58) | 4.66 (0.62) | 6.081E-131 | 2.6E-130 | 4.77E-06 | 1.14315E-05 | 0.322373 | 0.813063498 | 0 | 0 | 0 |
| Dkk.1 | 7.2 (0.39) | 8.06 (0.81) | 8.17 (0.74) | 6.09244E-85 | 8.27E-85 | 2.37E-08 | 8.03017E-08 | 0.257868 | 0.807608032 | 0 | 0 | 0 |
| LPL | 9.21 (0.58) | 9.09 (0.59) | 9.22 (0.71) | 4.09064E-27 | 4.25E-27 | 0.321976 | 0.364140061 | 0.143887 | 0.8914741 | 0 | 0 | 0 |
| PRSS8 | 8.17 (0.44) | 8.3 (0.66) | 8.4 (0.65) | 1.20582E-62 | 1.4E-62 | 0.312396 | 0.356131196 | 0.220186 | 0.814975774 | 0 | 0 | 0 |
| AGRP | 3.86 (0.4) | 5 (0.84) | 5.15 (0.82) | 1.204E-123 | 3.7E-123 | 3.68E-12 | 2.05827E-11 | 0.182979 | 0.802293999 | 0 | 0 | 0 |
| HB.EGF | 4.32 (0.34) | 4.72 (0.5) | 4.81 (0.48) | 5.5866E-123 | 1.6E-122 | 2.57E-05 | 5.30762E-05 | 0.177088 | 0.84116639 | 0 | 0 | 0 |
| GDF.2 | 7.46 (0.45) | 7.01 (0.77) | 7.16 (0.66) | 1.49721E-76 | 1.88E-76 | 0.00148 | 0.002510971 | 0.095243 | 0.848254553 | 0 | 0 | 0 |
| FABP2 | 7.64 (0.73) | 7.75 (1.33) | 8 (1.4) | 3.784E-58 | 4.28E-58 | 0.656041 | 0.700268773 | 0.16841 | 0.888832134 | 0 | 0 | 0 |
| THPO | 1.84 (0.34) | 2.18 (0.5) | 2.23 (0.64) | 5.0127E-142 | 5.3E-141 | 0.000314 | 0.000585146 | 0.471264 | 0.945846604 | 0 | 0 | 0 |
| MARCO | 6.63 (0.23) | 6.8 (0.42) | 6.84 (0.34) | 5.7729E-103 | 9.3E-103 | 0.026475 | 0.036275354 | 0.373734 | 0.852114235 | 0 | 0 | 0 |
| GT | 1.12 (0.51) | 2.04 (1.06) | 2.15 (1.14) | 5.5281E-136 | 3.4E-135 | 2.25E-06 | 5.7157E-06 | 0.46281 | 0.935467048 | 5.71 | 5.43 | 3.25 |
| MMP12 | 7.11 (0.59) | 8.02 (1.12) | 8.06 (1.2) | 1.36204E-74 | 1.7E-74 | 1.01E-05 | 2.20449E-05 | 0.804181 | 0.992170757 | 0 | 0 | 0 |
| ACE2 | 2.97 (0.53) | 3.97 (1.04) | 3.95 (1.12) | 1.9685E-122 | 5.6E-122 | 3.09E-07 | 9.06752E-07 | 0.906983 | 1 | 0 | 0 | 0 |
| PD.L2 | 2.82 (0.32) | 3.3 (0.53) | 3.33 (0.58) | 1.5574E-136 | 1E-135 | 2.17E-06 | 5.62384E-06 | 0.635849 | 0.9487794 | 0 | 0 | 0 |
| CTSL1 | 6.33 (0.32) | 7.29 (0.64) | 7.35 (0.62) | 4.7974E-102 | 7.6E-102 | 5.94E-14 | 4.23233E-13 | 0.482123 | 0.941131211 | 0 | 0 | 0 |
| hOSCAR | 10.61 (0.25) | 11.04 (0.32) | 11.03 (0.3) | 8.39176E-11 | 8.54E-11 | 1.03E-10 | 5.24342E-10 | 0.9048 | 1 | 0 | 0 | 0 |
| TNFRSF13B | 8.11 (0.29) | 8.55 (0.97) | 8.36 (1.02) | 2.87823E-72 | 3.49E-72 | 0.009144 | 0.013432888 | 0.142979 | 0.905534838 | 0 | 0 | 0 |
| TGM2 | 6.24 (0.65) | 6.93 (0.92) | 7.04 (1) | 6.97433E-84 | 9.38E-84 | 8.81E-05 | 0.000172042 | 0.3603 | 0.8557121 | 0 | 0 | 0 |
| LEP | 5.99 (1.22) | 6.51 (1.56) | 6.93 (1.34) | 8.30817E-62 | 9.59E-62 | 0.074032 | 0.095040966 | 0.025911 | 0.527477751 | 0 | 0 | 0 |
| HSP.27 | 8.87 (0.69) | 9.2 (0.51) | 9.32 (0.46) | 1.66717E-32 | 1.77E-32 | 0.004682 | 0.007372249 | 0.050189 | 0.572155754 | 0 | 0 | 0 |
| CD4 | 4.13 (0.26) | 5.11 (0.59) | 5.15 (0.67) | 1.2951E-129 | 5.1E-129 | 2.55E-16 | 2.34492E-15 | 0.702509 | 0.967223163 | 0 | 0 | 0 |
| NEMO | 4.11 (0.6) | 4.83 (0.71) | 5.01 (0.67) | 5.545E-109 | 1.1E-108 | 5.4E-07 | 1.47873E-06 | 0.040315 | 0.522268394 | 0 | 0 | 0 |
| VEGFD | 7.58 (0.37) | 7.32 (0.64) | 7.39 (0.55) | 1.53933E-79 | 1.99E-79 | 0.029721 | 0.040335618 | 0.364855 | 0.8453963 | 0 | 0 | 0 |
| HAOX1 | 4.35 (0.98) | 5.41 (2.16) | 5.77 (2.27) | 1.3367E-87 | 1.84E-87 | 0.006291 | 0.009638855 | 0.210752 | 0.845976349 | 0 | 0 | 0 |
| PPP1R9B | 3.09 (0.63) | 4.1 (0.88) | 4.23 (0.9) | 1.5198E-115 | 3.4E-115 | 6.45E-09 | 2.32794E-08 | 0.296781 | 0.821189764 | 0 | 0 | 0 |
| GLB1 | 2.42 (0.13) | 2.63 (0.39) | 2.63 (0.37) | 1.6141E-148 | 5.1E-147 | 0.002445 | 0.003982289 | 0.995568 | 0.999073549 | 37.14 | 21.74 | 19.48 |
| PSIP1 | 2 (0.62) | 2.65 (1.19) | 2.67 (1.08) | 6.1383E-124 | 1.9E-123 | 0.00249 | 0.004032541 | 0.866144 | 1 | 17.14 | 5.43 | 2.60 |
| ZBTB16 | 1.72 (0.59) | 2.46 (0.96) | 2.72 (0.92) | 2.5958E-127 | 9.4E-127 | 3.6E-05 | 7.27493E-05 | 0.03973 | 0.539195617 | 2.86 | 1.09 | 0 |
| TPSAB1 | 4.18 (0.71) | 3.83 (0.7) | 4.03 (0.78) | 1.4715E-102 | 2.4E-102 | 0.014892 | 0.021221133 | 0.043878 | 0.543703528 | 0 | 0 | 2.60 |
| HCLS1 | 3 (0.58) | 4.47 (0.84) | 4.47 (0.8) | 5.2094E-119 | 1.3E-118 | 2.11E-16 | 2.00327E-15 | 0.971864 | 1 | 0 | 0 | 0 |
| CLEC4G | 2.8 (0.31) | 3.53 (0.46) | 3.54 (0.64) | 2.4971E-137 | 1.9E-136 | 4.14E-14 | 3.02835E-13 | 0.878798 | 1 | 0 | 0 | 0 |
| CLEC4C | 3.89 (0.47) | 3.33 (1.01) | 3.27 (0.94) | 3.2799E-119 | 8.4E-119 | 0.002073 | 0.003454386 | 0.639604 | 0.949411515 | 0 | 4.35 | 6.49 |
| IRAK1 | 1.13 (0.42) | 1.84 (0.72) | 1.93 (0.67) | 1.5968E-141 | 1.6E-140 | 2.52E-07 | 7.56298E-07 | 0.32227 | 0.820061445 | 20.00 | 1.09 | 1.30 |
| CLEC4A | 3.94 (0.4) | 4.06 (0.52) | 4.06 (0.65) | 6.6988E-123 | 1.9E-122 | 0.224335 | 0.26419619 | 0.978552 | 1 | 0 | 0 | 0 |
| PRDX1 | 1.45 (0.25) | 1.86 (0.84) | 2.02 (1.01) | 2.1362E-149 | 7.6E-148 | 0.004772 | 0.007473069 | 0.211671 | 0.837866008 | 34.29 | 19.57 | 13.64 |
| PRDX5 | 3.89 (0.58) | 4.98 (0.85) | 5.27 (0.91) | 1.5936E-112 | 3.2E-112 | 1.42E-10 | 6.86389E-10 | 0.015432 | 0.488684877 | 0 | 0 | 0 |
| DPP10 | 1.28 (0.23) | 1.44 (0.59) | 1.52 (0.81) | 7.3116E-152 | 3.5E-150 | 0.121425 | 0.150461044 | 0.376716 | 0.845386797 | 25.71 | 22.83 | 22.73 |
| DCTN1 | 3.28 (0.63) | 3.96 (0.78) | 4.12 (0.76) | 4.4766E-114 | 9.3E-114 | 1.06E-05 | 2.3167E-05 | 0.095588 | 0.825528879 | 0 | 0 | 0 |
| ITGA6 | 1.63 (0.32) | 1.72 (0.32) | 1.76 (0.37) | 1.23E-144 | 1.8E-143 | 0.15687 | 0.188641615 | 0.315578 | 0.817634693 | 11.43 | 7.61 | 2.60 |
| CDSN | 2.67 (0.25) | 3.09 (0.68) | 3.11 (0.77) | 1.3355E-141 | 1.4E-140 | 0.000557 | 0.001005538 | 0.842754 | 1 | 28.57 | 21.74 | 23.38 |
| TRAF2 | 3.36 (0.55) | 3.32 (0.78) | 3.41 (0.7) | 1.7203E-118 | 4.3E-118 | 0.80425 | 0.833495441 | 0.352718 | 0.844745533 | 5.71 | 15.22 | 13.64 |
| TRIM21 | 1.75 (0.39) | 2.7 (0.76) | 2.73 (0.81) | 1.0833E-139 | 9.4E-139 | 1.39E-10 | 6.8203E-10 | 0.704033 | 0.960045396 | 22.86 | 2.17 | 2.60 |
| LILRB4 | 3.41 (0.5) | 5.09 (0.98) | 5.11 (0.85) | 2.8585E-121 | 8E-121 | 6.48E-17 | 6.83936E-16 | 0.861528 | 1 | 0 | 0 | 0 |
| NTF4 | 1.87 (0.36) | 1.75 (0.54) | 1.72 (0.45) | 8.7014E-141 | 8.3E-140 | 0.232374 | 0.271420854 | 0.681082 | 0.960933227 | 2.86 | 17.39 | 13.64 |
| KRT19 | 3.21 (0.83) | 4.55 (1.67) | 4.82 (1.57) | 4.5043E-104 | 7.5E-104 | 1.37E-05 | 2.90155E-05 | 0.192267 | 0.805823008 | 0 | 0 | 0.65 |
| ITM2A | 4.44 (0.57) | 3.9 (0.97) | 3.98 (0.98) | 6.6912E-108 | 1.3E-107 | 0.002586 | 0.00416413 | 0.512353 | 0.918368376 | 0 | 0 | 3.25 |
| HNMT | 8.52 (0.53) | 9.32 (0.97) | 9.66 (1.37) | 2.39014E-49 | 2.64E-49 | 9.02E-06 | 2.00887E-05 | 0.037808 | 0.538762993 | 0 | 0 | 0 |
| CCL11 | 7.28 (0.33) | 7.07 (0.62) | 7.16 (0.63) | 4.87391E-87 | 6.68E-87 | 0.062237 | 0.080625547 | 0.291864 | 0.823576722 | 0 | 0 | 0 |
| MILR1 | 3.5 (0.32) | 4.45 (0.75) | 4.58 (0.78) | 3.3319E-131 | 1.5E-130 | 4.31E-11 | 2.27677E-10 | 0.178654 | 0.83469672 | 0 | 0 | 0 |
| LY75 | 2.45 (0.24) | 2.53 (0.46) | 2.49 (0.38) | 8.2296E-144 | 9.8E-143 | 0.311146 | 0.356130855 | 0.539643 | 0.920947215 | 2.86 | 6.52 | 11.04 |
| EIF4G1 | 3.62 (0.8) | 4.69 (1.12) | 4.86 (0.98) | 3.8825E-102 | 6.1E-102 | 9.38E-07 | 2.52262E-06 | 0.219869 | 0.824507598 | 0 | 0 | 0 |
| HSD11B1 | 2.95 (0.45) | 2.64 (0.61) | 2.65 (0.56) | 3.0602E-127 | 1.1E-126 | 0.007767 | 0.011588802 | 0.933891 | 1 | 0 | 0 | 1.95 |
| PLXNA4 | 5.68 (0.8) | 5.28 (1.06) | 5.45 (1) | 5.77018E-83 | 7.68E-83 | 0.04559 | 0.060153438 | 0.22926 | 0.827077375 | 0 | 0 | 0 |
| SH2B3 | 3.05 (1) | 3.55 (0.69) | 3.66 (0.77) | 2.53568E-97 | 3.71E-97 | 0.001588 | 0.002662611 | 0.288834 | 0.823175628 | 31.43 | 1.09 | 1.95 |
| CKAP4 | 3.92 (0.4) | 5.55 (0.78) | 5.68 (0.9) | 4.7721E-123 | 1.4E-122 | 6.46E-22 | 1.41585E-20 | 0.245717 | 0.80493415 | 0 | 0 | 0 |
| HEXIM1 | 4.61 (0.46) | 5.71 (0.95) | 5.88 (0.8) | 6.3642E-113 | 1.3E-112 | 1.23E-09 | 4.85108E-09 | 0.129204 | 0.898126599 | 0 | 0 | 0 |
| CLEC4D | 1.79 (0.44) | 4.26 (1.35) | 4.17 (1.41) | 4.1527E-136 | 2.6E-135 | 3.92E-19 | 5.07429E-18 | 0.629573 | 0.959509107 | 0 | 0 | 0 |
| MGMT | 2.79 (0.68) | 3.84 (1.08) | 3.97 (1.21) | 7.2395E-115 | 1.6E-114 | 4.2E-07 | 1.18597E-06 | 0.393048 | 0.861682185 | 0 | 0 | 1.30 |
| TREM1 | 0.03 (0.12) | 0.99 (0.73) | 0.96 (0.71) | 2.2186E-163 | 6.3E-161 | 2.34E-12 | 1.33567E-11 | 0.699508 | 0.967766509 | 0 | 0 | 0 |
| CXADR | 1.66 (0.34) | 2.62 (0.88) | 2.74 (0.95) | 6.6126E-143 | 7.5E-142 | 5.06E-09 | 1.87179E-08 | 0.32967 | 0.824175721 | 0 | 0 | 0 |
| IL10 | 3.4 (0.48) | 7.08 (2.01) | 7.2 (2.89) | 2.1329E-122 | 6E-122 | 2.45E-19 | 3.49518E-18 | 0.717955 | 0.974367181 | 0 | 0 | 0 |
| KLRD1 | 6.51 (0.65) | 6.98 (0.76) | 7.04 (0.79) | 1.73275E-80 | 2.27E-80 | 0.001451 | 0.002476283 | 0.551857 | 0.930645741 | 0 | 0 | 0 |
| PIK3AP1 | 3.11 (0.41) | 4.31 (1.1) | 4.24 (1.03) | 3.8831E-129 | 1.5E-128 | 4.23E-09 | 1.58606E-08 | 0.611006 | 0.956795544 | 2.86 | 0 | 0.65 |
| STC1 | 6.45 (0.38) | 7.35 (0.37) | 7.3 (0.37) | 3.45747E-97 | 5.03E-97 | 4.08E-23 | 1.1626E-21 | 0.343878 | 0.830552226 | 0 | 0 | 0 |
| FAM3B | 4.35 (0.45) | 4.41 (0.81) | 4.55 (0.78) | 2.688E-116 | 6E-116 | 0.662878 | 0.702306092 | 0.186067 | 0.803471806 | 0 | 0 | 0 |
| SH2D1A | 1.95 (0.41) | 2 (0.75) | 2.03 (0.56) | 6.8097E-137 | 4.6E-136 | 0.664039 | 0.700930015 | 0.72859 | 0.979471891 | 11.43 | 15.22 | 12.34 |
| DFFA | 4.28 (0.29) | 5.42 (1) | 5.51 (0.98) | 1.7333E-126 | 6E-126 | 8.92E-10 | 3.63016E-09 | 0.486972 | 0.937749781 | 0 | 0 | 0 |
| DCBLD2 | 8.38 (0.27) | 8.11 (0.57) | 8.25 (0.54) | 1.11365E-66 | 1.33E-66 | 0.009517 | 0.013909756 | 0.068378 | 0.695995401 | 0 | 0 | 0 |
| FCRL6 | 4.03 (0.68) | 4.54 (0.81) | 4.54 (0.81) | 4.721E-105 | 8.2E-105 | 0.001296 | 0.002237994 | 0.982272 | 1 | 0 | 0 | 0 |
| NCR1 | 2.01 (0.35) | 2.53 (0.63) | 2.63 (0.69) | 4.5841E-140 | 4.1E-139 | 8.29E-06 | 1.90466E-05 | 0.232986 | 0.809768452 | 0 | 0 | 0 |
| AREG | 2.56 (0.44) | 4.21 (1.39) | 4.3 (1.52) | 4.6578E-131 | 2.1E-130 | 2.79E-10 | 1.26255E-09 | 0.621915 | 0.952934738 | 0 | 0 | 0 |
| IFNLR1 | 2.28 (0.37) | 2.5 (0.43) | 2.48 (0.43) | 2.3378E-137 | 1.8E-136 | 0.007822 | 0.011611321 | 0.769565 | 0.987955132 | 0 | 0 | 0 |
| SIT1 | 2.92 (0.44) | 2.81 (0.72) | 2.75 (0.63) | 1.5705E-128 | 5.7E-128 | 0.401813 | 0.442149712 | 0.509756 | 0.925354076 | 2.86 | 0 | 1.95 |
| MASP1 | 1.91 (0.26) | 2.09 (0.36) | 2.12 (0.41) | 5.622E-146 | 1.1E-144 | 0.0061 | 0.009397539 | 0.611738 | 0.952706755 | 0 | 0 | 0 |
| LAMP3 | 4.2 (0.71) | 4.76 (0.82) | 4.71 (0.75) | 1.7187E-102 | 2.7E-102 | 0.000504 | 0.000920669 | 0.630335 | 0.955560367 | 0 | 0 | 0 |
| CLEC7A | 2.84 (0.5) | 3.69 (0.86) | 3.68 (0.94) | 1.8246E-125 | 5.9E-125 | 1.78E-07 | 5.45774E-07 | 0.931984 | 1 | 0 | 0 | 0.65 |
| CLEC6A | 2.24 (0.38) | 3.97 (0.84) | 3.85 (0.91) | 3.8489E-137 | 2.7E-136 | 6.66E-22 | 1.35661E-20 | 0.279017 | 0.811427791 | 5.71 | 0 | 0 |
| DDX58 | 2.2 (0.33) | 2.94 (0.86) | 2.86 (0.86) | 2.0959E-140 | 1.9E-139 | 2.46E-06 | 6.1998E-06 | 0.506962 | 0.932155438 | 11.43 | 1.09 | 2.60 |
| ITGA11 | 2.15 (0.48) | 1.8 (0.66) | 1.76 (0.48) | 4.4891E-131 | 2E-130 | 0.000766 | 0.001355338 | 0.528799 | 0.924587209 | 2.86 | 11.96 | 15.58 |
| LAG3 | 2.74 (0.33) | 3.19 (0.93) | 3.14 (0.8) | 2.9022E-136 | 1.8E-135 | 0.005435 | 0.008463616 | 0.659842 | 0.94977188 | 2.86 | 2.17 | 2.60 |
| CD83 | 3.04 (0.36) | 3.38 (0.83) | 3.41 (0.71) | 3.9126E-132 | 2E-131 | 0.022439 | 0.031044742 | 0.741598 | 0.98304914 | 0 | 0 | 0 |
| ITGB6 | 2.87 (0.31) | 2.79 (0.66) | 2.79 (0.53) | 7.657E-137 | 5.1E-136 | 0.479221 | 0.521289514 | 0.985317 | 1 | 0 | 0 | 0 |
| BTN3A2 | 2.94 (0.28) | 4.32 (0.72) | 4.39 (0.86) | 1.1007E-137 | 9E-137 | 4.79E-20 | 8.02596E-19 | 0.560219 | 0.92826909 | 0 | 0 | 0 |
| IL8 | 4.55 (0.37) | 7.62 (2.09) | 7.53 (2.29) | 2.8356E-119 | 7.3E-119 | 2.5E-14 | 1.87159E-13 | 0.755769 | 0.979064797 | 0 | 0 | 0 |
| VEGFA | 9.98 (0.29) | 11.49 0.69) | 11.45 (0.63) | 2.29057E-06 | 2.31E-06 | 8.32E-24 | 2.96307E-22 | 0.667211 | 0.955553987 | 0 | 0 | 0 |
| CD8A | 9.79 (0.45) | 10.44 (0.77) | 10.33 (0.97) | 1.02789E-10 | 1.04E-10 | 7.12E-06 | 1.67763E-05 | 0.372677 | 0.856556963 | 0 | 0 | 0 |
| CDCP1 | 2.63 (0.54) | 3.86 (0.86) | 3.79 (0.65) | 4.4144E-124 | 1.4E-123 | 1.32E-12 | 7.84135E-12 | 0.477311 | 0.951284395 | 0 | 0 | 0 |
| CD244 | 6.46 (0.35) | 6.46 (0.52) | 6.45 (0.49) | 1.63787E-98 | 2.43E-98 | 0.963696 | 0.963695652 | 0.920444 | 1 | 0 | 0 | 0 |
| IL7 | 2.21 (0.44) | 2.7 (0.51) | 2.81 (0.52) | 1.3408E-133 | 7.2E-133 | 1.74E-06 | 4.54778E-06 | 0.10595 | 0.816101857 | 0 | 0 | 0 |
| OPG | 9.61 (0.31) | 11.01 (0.84) | 10.96 (0.76) | 2.95692E-21 | 3.03E-21 | 1.23E-16 | 1.2108E-15 | 0.609036 | 0.958979441 | 0 | 0 | 0 |
| LAP.TGF.beta.1 | 6.79 (0.23) | 7.64 (0.52) | 7.64 (0.42) | 2.1528E-100 | 3.3E-100 | 3.53E-16 | 3.13951E-15 | 0.991463 | 1 | 0 | 0 | 0 |
| uPA | 10.03 (0.2) | 9.91 (0.46) | 9.87 (0.43) | 3.62267E-05 | 3.65E-05 | 0.139572 | 0.169268711 | 0.487132 | 0.93176272 | 0 | 0 | 0 |
| MCP.1 | 11.06 (0.35) | 12.66 (1.37) | 12.24 (1.32) | 1.47865E-27 | 1.55E-27 | 4.09E-10 | 1.79138E-09 | 0.017573 | 0.455312254 | 0 | 0 | 0 |
| CXCL11 | 7.46 (0.52) | 8.59 (1.45) | 8.34 (1.29) | 1.09496E-72 | 1.34E-72 | 1.58E-05 | 3.31341E-05 | 0.158605 | 0.886320766 | 0 | 0 | 0 |
| AXIN1 | 2.24 (0.67) | 3.13 (0.79) | 3.25 (0.67) | 1.7536E-119 | 4.6E-119 | 3.23E-08 | 1.08229E-07 | 0.229855 | 0.818859063 | 0 | 0 | 0 |
| TRAIL | 7.83 (0.24) | 6.75 (0.72) | 6.75 (0.68) | 4.13883E-81 | 5.44E-81 | 1.94E-14 | 1.49515E-13 | 0.990622 | 1 | 0 | 0 | 0 |
| CXCL9 | 7.03 (0.77) | 8.43 (1.48) | 8.34 (1.37) | 1.17297E-66 | 1.39E-66 | 4.48E-07 | 1.25314E-06 | 0.632377 | 0.953584993 | 0 | 0 | 0 |
| CST5 | 5.09 (0.45) | 5.07 (0.73) | 5.19 (0.65) | 1.0969E-108 | 2.1E-108 | 0.877999 | 0.903356409 | 0.180511 | 0.816596414 | 0 | 0 | 0 |
| OSM | 2.11 (0.46) | 6.22 (1.41) | 5.72 (1.59) | 5.5974E-133 | 3E-132 | 5.58E-34 | 5.30161E-32 | 0.013735 | 0.652412952 | 0 | 0 | 0 |
| CCL4 | 5.29 (0.56) | 6.87 (1.62) | 6.97 (1.97) | 5.0334E-100 | 7.6E-100 | 1.04E-07 | 3.36844E-07 | 0.685085 | 0.96181853 | 0 | 0 | 0 |
| CD6 | 5.17 (0.43) | 4.8 (0.83) | 4.73 (0.76) | 1.3073E-109 | 2.5E-109 | 0.014023 | 0.02018516 | 0.517184 | 0.921234487 | 0 | 0 | 0 |
| TGF.alpha | 2.37 (0.24) | 4.57 (1.04) | 4.39 (1.09) | 3.7398E-144 | 4.6E-143 | 2E-23 | 6.32897E-22 | 0.214554 | 0.815306495 | 0 | 0 | 0.65 |
| MCP.4 | 12.94 (0.41) | 12.67 (0.95) | 12.77 (0.87) | 3.23741E-77 | 4.12E-77 | 0.114043 | 0.141931014 | 0.389286 | 0.860050436 | 0 | 0 | 0 |
| TNFSF14 | 3.31 (0.42) | 5.39 (1) | 5.32 (0.87) | 1.3855E-126 | 4.9E-126 | 2.66E-22 | 6.31799E-21 | 0.58391 | 0.945535316 | 0 | 0 | 0 |
| IL.10RA | 1.02 (0.5) | 1.13 (0.65) | 1.15 (0.56) | 3.2125E-137 | 2.3E-136 | 0.397867 | 0.439504605 | 0.77868 | 0.986328619 | 22.86 | 11.96 | 7.79 |
| MMP.1 | 7.47 (0.76) | 8.89 (1.23) | 8.93 (1.43) | 5.81453E-60 | 6.63E-60 | 3.78E-09 | 1.43651E-08 | 0.791778 | 0.985401073 | 0 | 0 | 0 |
| LIF.R | 3.5 (0.25) | 4.05 (0.43) | 4.08 (0.42) | 1.9641E-135 | 1.2E-134 | 8.23E-11 | 4.26393E-10 | 0.571103 | 0.935427209 | 0 | 0 | 0 |
| CCL19 | 8.1 (0.79) | 10 (1.26) | 9.7 (1.15) | 8.23607E-47 | 9.06E-47 | 1.35E-13 | 9.16341E-13 | 0.060021 | 0.657923197 | 0 | 0 | 0 |
| IL.15RA | 1.02 (0.24) | 1.5 (0.61) | 1.53 (0.61) | 1.9859E-152 | 1.1E-150 | 1.73E-05 | 3.59825E-05 | 0.764306 | 0.985643034 | 0 | 2.17 | 0 |
| IL.10RB | 5.47 (0.28) | 5.87 (0.39) | 5.93 (0.42) | 6.3175E-115 | 1.4E-114 | 1.88E-07 | 5.69898E-07 | 0.263556 | 0.807671293 | 0 | 0 | 0 |
| IL.18R1 | 7.82 (0.37) | 8.69 (0.72) | 8.69 (0.73) | 1.47056E-74 | 1.82E-74 | 5.13E-10 | 2.1839E-09 | 0.982215 | 1 | 0 | 0 | 0 |
| PD.L1 | 6.05 (0.31) | 6.95 (0.75) | 6.92 (0.68) | 1.1929E-106 | 2.2E-106 | 2.12E-10 | 9.90955E-10 | 0.720111 | 0.972662077 | 0 | 0 | 0 |
| CXCL5 | 8.83 (0.88) | 8.82 (1.15) | 9.08 (1.17) | 3.49636E-27 | 3.65E-27 | 0.957858 | 0.961230801 | 0.097773 | 0.796151853 | 0 | 0 | 0 |
| TRANCE | 4.35 (0.62) | 2.45 (0.85) | 2.45 (0.69) | 6.1673E-106 | 1.1E-105 | 9.04E-23 | 2.34347E-21 | 0.983644 | 1 | 0 | 3.26 | 0 |
| HGF | 7.98 (0.3) | 9.78 (0.98) | 9.79 (0.89) | 6.37697E-75 | 7.97E-75 | 2.74E-19 | 3.72004E-18 | 0.964966 | 1 | 0 | 0 | 0 |
| IL.12B | 5.19 (0.52) | 5.95 (1.22) | 5.73 (1.22) | 1.2453E-103 | 2.1E-103 | 0.000516 | 0.00093711 | 0.171402 | 0.857009867 | 0 | 0 | 0 |
| MMP.10 | 8 (0.6) | 8.9 (0.93) | 9.08 (1.02) | 1.09143E-57 | 1.23E-57 | 4.91E-07 | 1.35767E-06 | 0.161515 | 0.885224991 | 0 | 0 | 0 |
| TNF | 2.56 (0.45) | 4.12 (1.63) | 4.51 (2.14) | 1.526E-130 | 6.5E-130 | 1.4E-07 | 4.32687E-07 | 0.13265 | 0.900126975 | 0 | 0 | 0 |
| CCL23 | 9.19 (0.3) | 11.08 (0.74) | 11.12 (0.69) | 2.26052E-39 | 2.42E-39 | 1.02E-28 | 4.82961E-27 | 0.647388 | 0.951059053 | 0 | 0 | 0 |
| CD5 | 4.65 (0.36) | 4.94 (0.77) | 4.88 (0.68) | 4.66E-119 | 1.2E-118 | 0.032027 | 0.042652728 | 0.508663 | 0.929288323 | 0 | 0 | 0 |
| Flt3L | 9.02 (0.35) | 8.67 (0.82) | 8.65 (0.96) | 3.89612E-43 | 4.22E-43 | 0.017911 | 0.025145804 | 0.880425 | 1 | 0 | 0 | 0 |
| CXCL6 | 7.51 (0.51) | 8.02 (0.81) | 8.14 (0.94) | 2.22261E-72 | 2.71E-72 | 0.000852 | 0.001499649 | 0.301738 | 0.811276538 | 0 | 0 | 0 |
| CXCL10 | 9.05 (0.72) | 10.93 (1.57) | 10.47 (1.52) | 6.49042E-27 | 6.7E-27 | 3.77E-10 | 1.67839E-09 | 0.024278 | 0.532247383 | 0 | 0 | 0 |
| X4E.BP1 | 5.84 (0.62) | 7.47 (1.46) | 7.65 (1.37) | 1.61907E-90 | 2.25E-90 | 3.38E-09 | 1.30008E-08 | 0.337497 | 0.829194714 | 0 | 0 | 0 |
| SIRT2 | 2.6 (0.66) | 3.65 (1.28) | 3.86 (1.19) | 1.5626E-117 | 3.7E-117 | 8.7E-06 | 1.96859E-05 | 0.204723 | 0.833516746 | 20.00 | 2.17 | 1.30 |
| CCL28 | 2.04 (0.39) | 2.24 (0.4) | 2.4 (0.41) | 1.9692E-137 | 1.6E-136 | 0.016506 | 0.023403563 | 0.00312 | 0.296380045 | 0 | 0 | 0 |
| DNER | 8.64 (0.21) | 8.08 (0.38) | 8.07 (0.37) | 4.36628E-62 | 5.06E-62 | 3.83E-13 | 2.4256E-12 | 0.819707 | 0.99411299 | 0 | 0 | 0 |
| EN.RAGE | 2.97  (0.67) | 4.88 (1.09) | 4.91 (1.12) | 1.7795E-114 | 3.8E-114 | 8.99E-17 | 9.14758E-16 | 0.840385 | 1 | 0 | 0 | 0 |
| CD40 | 11.09 (0.39) | 11.82 (0.8) | 11.9 (0.71) | 5.2481E-27 | 5.44E-27 | 9.16E-07 | 2.48594E-06 | 0.412451 | 0.858019014 | 0 | 0 | 0 |
| IFN.gamma | 6.07 (1.07) | 8.54 (3.07) | 7.84 (2.89) | 3.06357E-66 | 3.62E-66 | 8.79E-06 | 1.97254E-05 | 0.075968 | 0.746586819 | 0 | 0 | 3.90 |
| FGF.19 | 7.94 (0.86) | 8.26 (1.3) | 8.4(1.32) | 8.86067E-47 | 9.71E-47 | 0.173904 | 0.206510811 | 0.431133 | 0.883977 | 0 | 0 | 0 |
| MCP.2 | 8.03 (0.48) | 9.25 (1.42) | 9.16 (1.65) | 1.08699E-63 | 1.27E-63 | 2.49E-06 | 6.23056E-06 | 0.677331 | 0.960394969 | 0 | 0 | 0 |
| CCL25 | 5.73 (0.45) | 5.83 (0.78) | 5.96 (0.8) | 4.9297E-102 | 7.7E-102 | 0.488228 | 0.529068546 | 0.213846 | 0.823595505 | 0 | 0 | 0 |
| CX3CL1 | 5.37 (0.3) | 5.89 (0.68) | 6.03 (0.83) | 3.4923E-115 | 7.7E-115 | 3.17E-05 | 6.50232E-05 | 0.169236 | 0.86129185 | 0 | 0 | 0 |
| TNFRSF9 | 6.52 (0.46) | 7.36 (1.21) | 7.36 (1.11) | 2.00765E-91 | 2.83E-91 | 0.000104 | 0.000202021 | 0.969139 | 1 | 0 | 0 | 0 |
| NT.3 | 2.26 (0.48) | 2.05 (0.57) | 1.97 (0.58) | 1.7366E-130 | 7.3E-130 | 0.054612 | 0.071396806 | 0.244606 | 0.81061162 | 0 | 5.43 | 5.84 |
| TWEAK | 8.97 (0.23) | 8.37 (0.49) | 8.39 (0.38) | 2.39287E-50 | 2.65E-50 | 2.69E-10 | 1.23449E-09 | 0.781883 | 0.981659593 | 0 | 0 | 0 |
| CCL20 | 6.14 (1.17) | 9.64 (1.84) | 9.44 (1.72) | 1.00247E-61 | 1.15E-61 | 7.68E-19 | 9.51092E-18 | 0.409873 | 0.858925934 | 0 | 0 | 0 |
| ST1A1 | 2.91 (0.83) | 3.05 (0.72) | 3.37 (0.83) | 4.1851E-106 | 7.5E-106 | 0.333612 | 0.374328505 | 0.00298 | 0.849253249 | 11.43 | 1.09 | 0 |
| STAMBP | 3.86 (0.55) | 4.56 (1) | 4.65 (0.93) | 2.8623E-114 | 6E-114 | 0.000133 | 0.000250941 | 0.501368 | 0.940064935 | 0 | 0 | 0 |
| ADA | 4.94 (0.29) | 5.18 (0.9) | 5.05 (0.61) | 2.3975E-120 | 6.4E-120 | 0.125546 | 0.154226962 | 0.176524 | 0.852700367 | 0 | 0 | 0 |
| TNFB | 4.42 (0.46) | 3.97 (0.93) | 3.84 (0.81) | 6.5333E-115 | 1.4E-114 | 0.008052 | 0.01189093 | 0.232973 | 0.819720191 | 0 | 0 | 0 |
| CSF.1 | 9.46 (0.21) | 10.25 (0.27) | 10.29 (0.26) | 1.59344E-31 | 1.68E-31 | 1.59E-31 | 1.13533E-29 | 0.243601 | 0.816780671 | 0 | 0 | 0 |
