# Supplementary material for: Identification of protein biomarkers to differentiate between gram-negative and gram-positive infections in adults suspected of sepsis
Source: BMC Infect Dis. 2025 Nov 14;25:1576. doi: 10.1186/s12879-025-11973-5 (PMC12619434; doi:10.1186/s12879-025-11973-5)
Supplement: Supplementary file 5 — Supplementary Material 5: A ranked list of positive and negative coefficients of discriminative proteins selected by Lasso [file 12879_2025_11973_MOESM5_ESM.docx]

Additional file 5. A ranked list of positive and negative coefficients of discriminative proteins selected by the Lasso algorithm. Red indicates a positive coefficient and blue indicates a negative.

| **Protein** | **Lasso Coefficient** | **PEA Panel** |
| --- | --- | --- |
| IL-1Ra | 0.099082 | CVDII^1^ |
| PRDX5 | 0.089517 | IR^2^ |
| KIM1 | 0.071584 | CVDII |
| CCL28 | 0.061611 | Inf^3^ |
| 4E-BP1 | 0.05655 | Inf |
| CSF-1 | 0.054647 | Inf |
| IL-6 | 0.050734 | CVDII, IR, Inf |
| ST1A1 | 0.044502 | Inf |
| VSIG2 | 0.043475 | CVDII |
| LEP | 0.040256 | CVDII |
| CXCL9 | 0.037211 | Inf |
| TNF | 0.028083 | Inf |
| IL10 | 0.027703 | IR, Inf |
| FGF-21 | 0.021661 | CVDII, Inf |
| MMP-10 | 0.020631 | Inf |
| CFHR5 | 0.020095 | CM^4^ |
| EN.RAGE | 0.019807 | Inf |
| NCR1 | 0.018277 | IR |
| TPSAB1 | 0.017578 | IR |
| HAOX1 | 0.016773 | CVDII |
| MILR1 | 0.014805 | IR |
| ANG | 0.012596 | CM |
| MCP-4 | 0.012575 | Inf |
| SAA4 | 0.011647 | CM |
| MBL2 | 0.010684 | CM |
| NEMO | 0.010644 | CVDII |
| BMP-6 | 0.008998 | CVDII |
| CCL25 | 0.008581 | Inf |
| MFAP5 | 0.008314 | CM |
| REN | 0.00806 | CVDII |
| GDF-2 | 0.00354 | CVDII |
| MCP-2 | 0.000691 | Inf |
| MMP-1 | 0.000157 | Inf |
| IFN-*γ* | -0.00135 | Inf |
| SELL | -0.00416 | CM |
| TRAF2 | -0.00447 | IR |
| TIMD4 | -0.00547 | CM |
| GIF | -0.00607 | CVDII |
| CD8A | -0.00779 | Inf |
| TNFB | -0.00873 | Inf |
| CCL19 | -0.01364 | Inf |
| TNFRSF13B | -0.01543 | CVDII |
| CCL17 | -0.02415 | CVDII |
| IDUA | -0.03197 | CVDII |
| IL-12B | -0.03769 | Inf |
| ACE2 | -0.03822 | CVDII |
| FGF-23 | -0.04052 | CVDII, Inf |
| ADA | -0.04753 | Inf |
| CEACAM8 | -0.05122 | CVDII |
| IL7R | -0.05228 | CM |
| TRANCE | -0.06551 | Inf |
| CNDP1 | -0.07275 | CM |
| GLO1 | -0.08438 | CVDII |
| COMP | -0.10757 | CM |
| MCP-1 | -0.18666 | Inf |

^1^Cardiovascular II; CVDII, ^2^ Immune response; IR, ^3^ Inflammation; Inf, ^4^ Cardiomatabolic; CM
