# Supplementary figures and images for: Identification of protein biomarkers to differentiate between gram-negative and gram-positive infections in adults suspected of sepsis
Source: BMC Infect Dis. 2025 Nov 14;25:1576. doi: 10.1186/s12879-025-11973-5 (PMC12619434; doi:10.1186/s12879-025-11973-5)

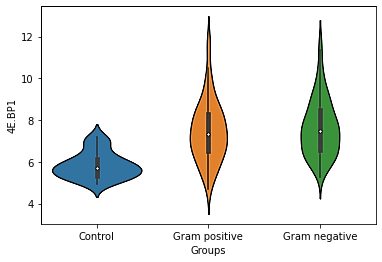

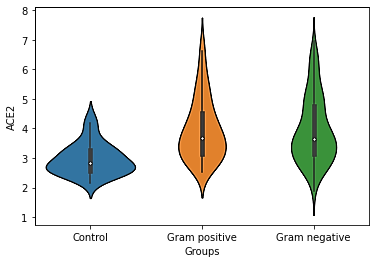

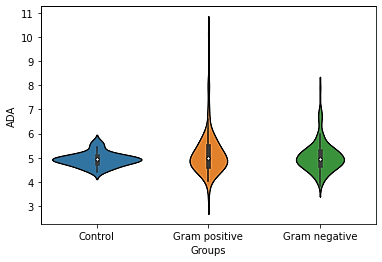

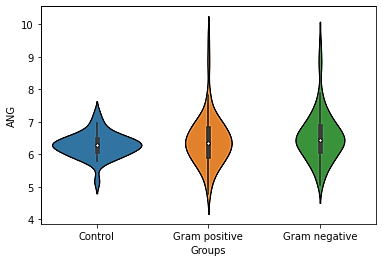

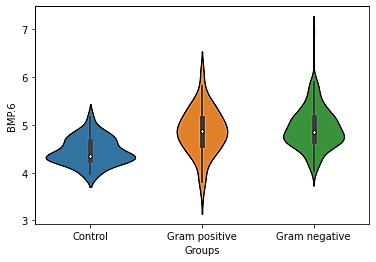

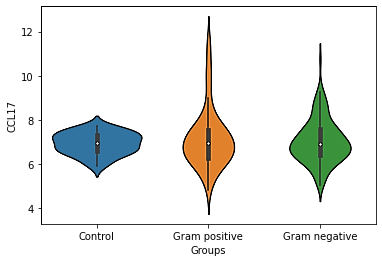

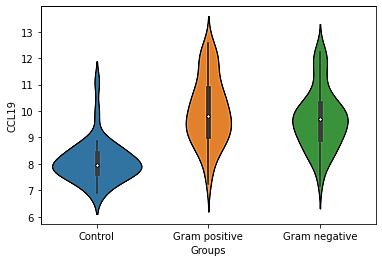

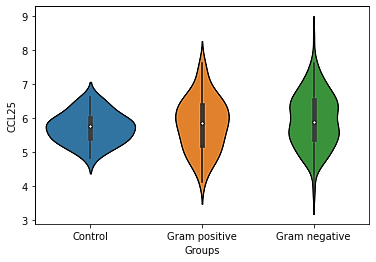

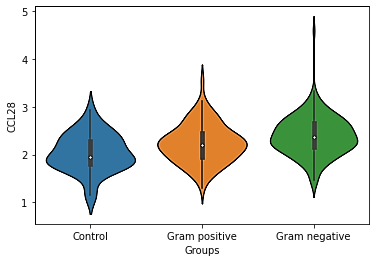

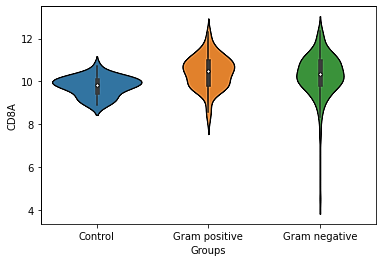

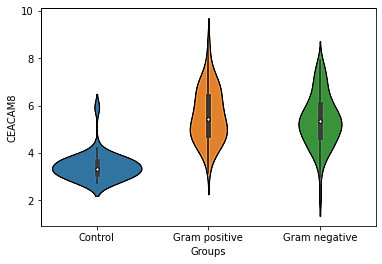

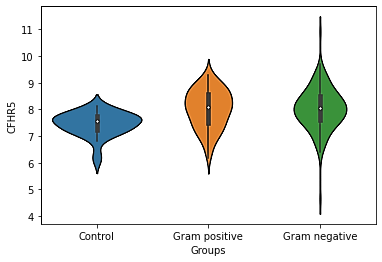

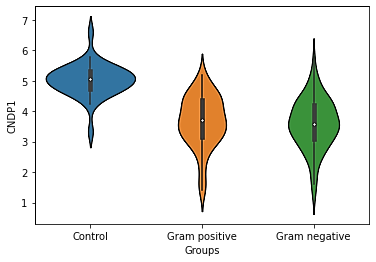

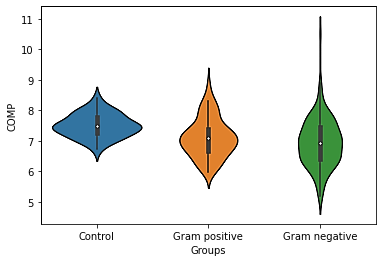

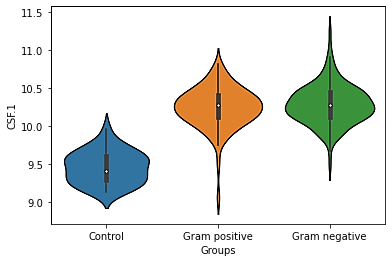

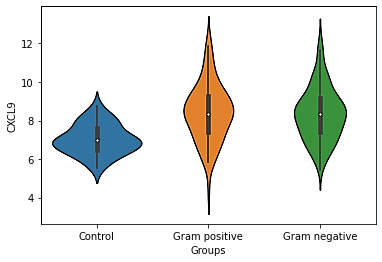

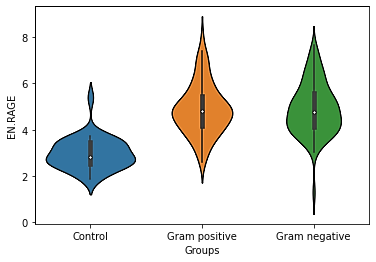

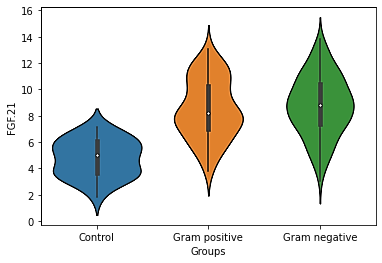

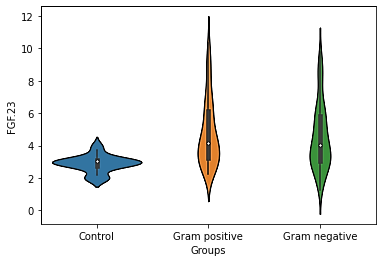

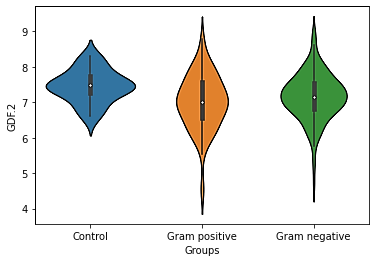

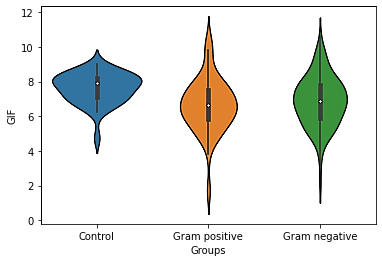

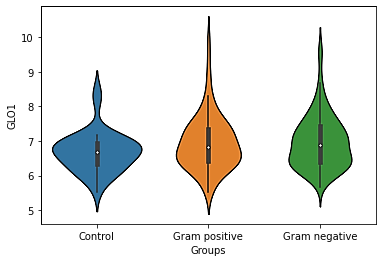

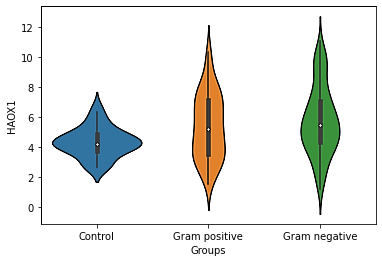

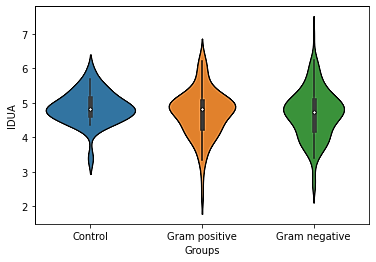

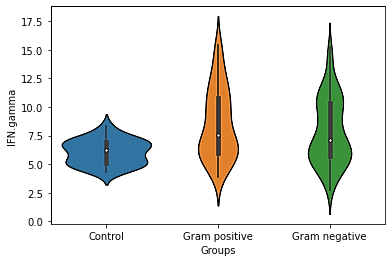

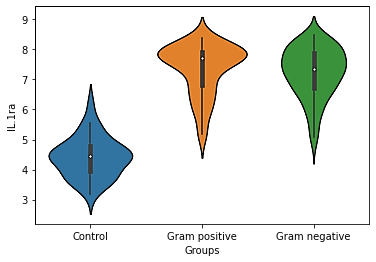

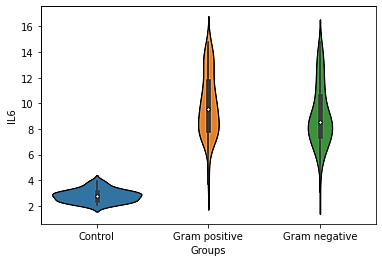

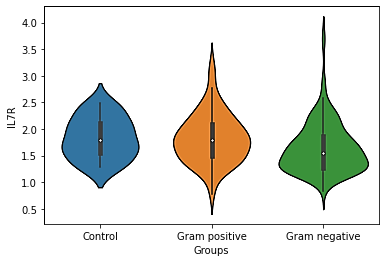

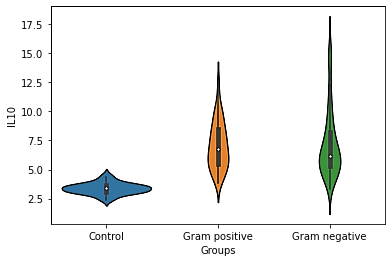

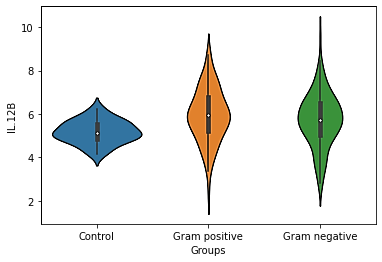

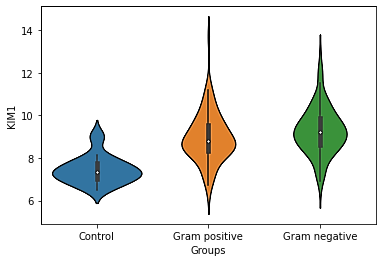

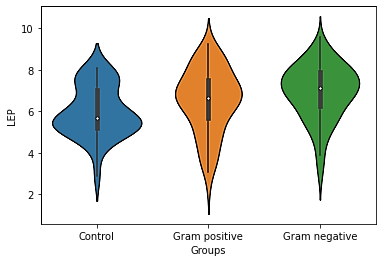

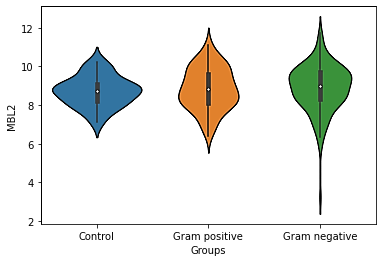

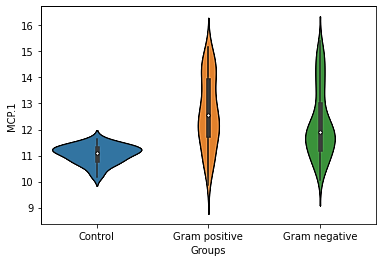

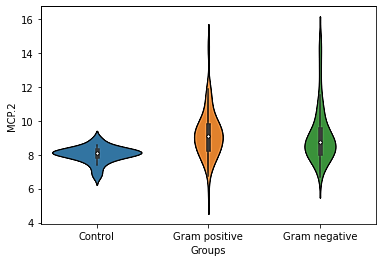

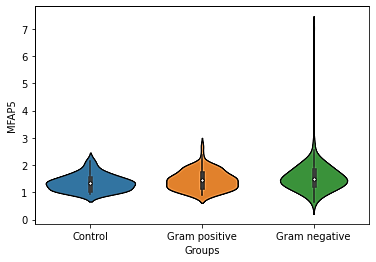

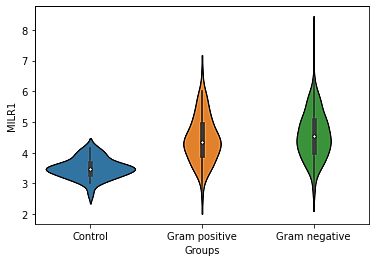

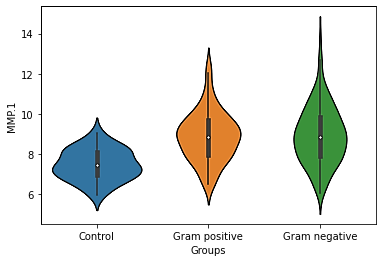

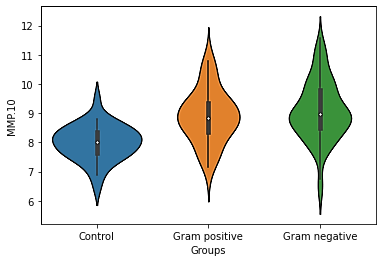

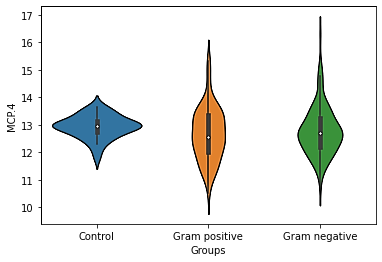

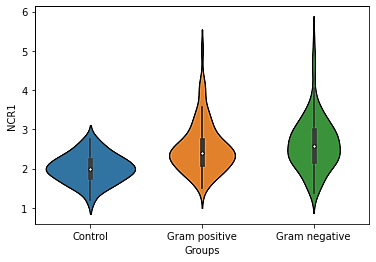

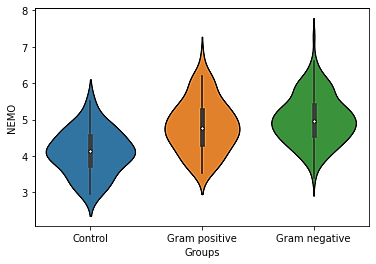

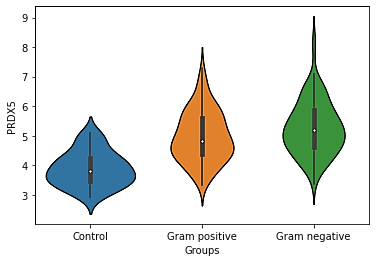

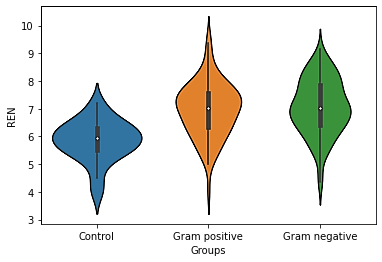

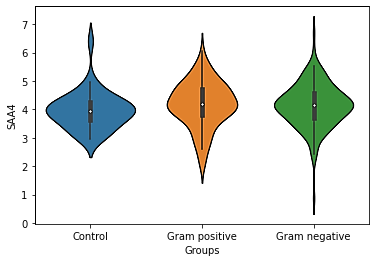

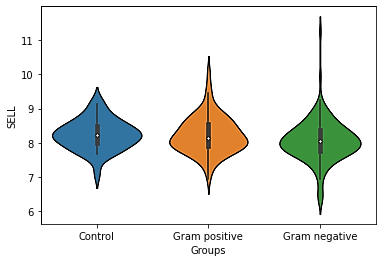

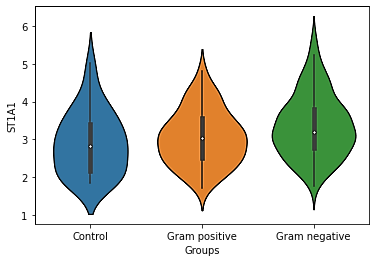

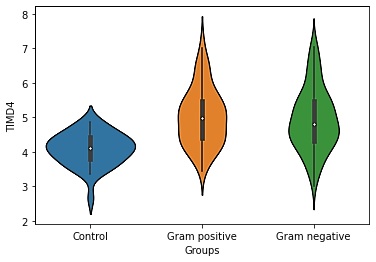

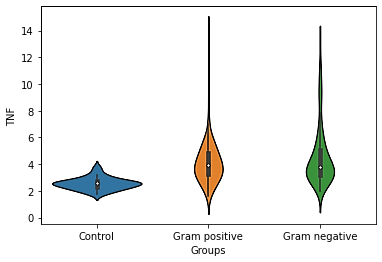

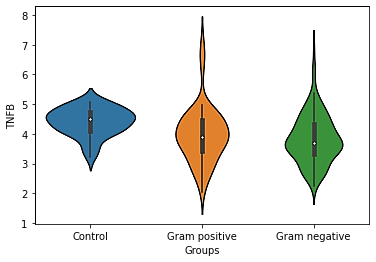

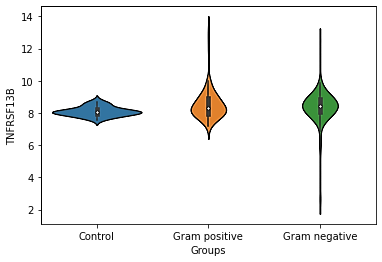

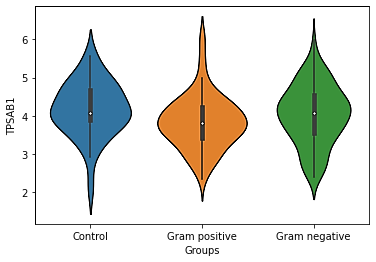

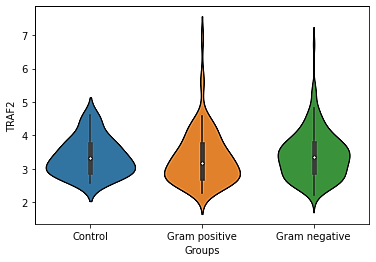

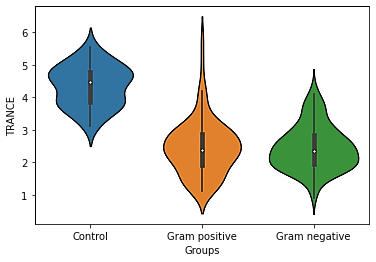

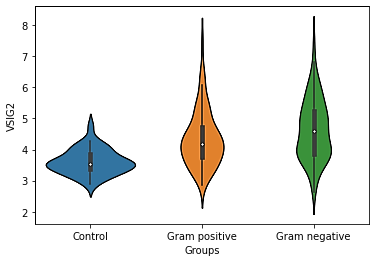


Additional file 7. Violin plot of 55 predictive protein.

Supplement: Supplementary file 6 — Supplementary Material 6: Violen plot of 55 discriminative proteins [file 12879_2025_11973_MOESM6_ESM.docx]
